# A Review on the Molecular Mechanisms of Action of Natural Products in Preventing Bone Diseases

**DOI:** 10.3390/ijms23158468

**Published:** 2022-07-30

**Authors:** Innocent U. Okagu, Timothy P. C. Ezeorba, Rita N. Aguchem, Ikenna C. Ohanenye, Emmanuel C. Aham, Sunday N. Okafor, Carlotta Bollati, Carmen Lammi

**Affiliations:** 1 Department of Biochemistry, Faculty of Biological Sciences, University of Nigeria, Nsukka 410001, Nigeria; innocent.okagu@unn.edu.ng (I.U.O.); timothy.ezeorba@unn.edu.ng (T.P.C.E.); aguchemrita@gmail.com (R.N.A.); emmanuel.aham@unn.edu.ng (E.C.A.); 2 School of Nutrition Sciences, Faculty of Health Sciences, University of Ottawa, Ottawa, ON K1H 8M5, Canada; iohaneny@uottawa.ca; 3 Natural Science Unit, School of General Studies, University of Nigeria, Nsukka 410001, Nigeria; 4 School of Environment and Safety Engineering, Jiangsu University, Zhenjiang 212013, China; 5 Department of Pharmaceutical and Medicinal Chemistry, University of Nigeria, Nsukka 410001, Nigeria; sunday.okafor@unn.edu.ng; 6 Department of Pharmaceutical Sciences, University of Milan, via Mangiagalli 25, 20133 Milano, Italy; carlotta.bollati@unimi.it

**Keywords:** bioactive compounds, bone diseases, bone remodeling, natural products, osteoprotective properties, bone signaling pathways

## Abstract

The drugs used for treating bone diseases (BDs), at present, elicit hazardous side effects that include certain types of cancers and strokes, hence the ongoing quest for the discovery of alternatives with little or no side effects. Natural products (NPs), mainly of plant origin, have shown compelling promise in the treatments of BDs, with little or no side effects. However, the paucity in knowledge of the mechanisms behind their activities on bone remodeling has remained a hindrance to NPs’ adoption. This review discusses the pathological development of some BDs, the NP-targeted components, and the actions exerted on bone remodeling signaling pathways (e.g., Receptor Activator of Nuclear Factor κ B-ligand (RANKL)/monocyte/macrophage colony-stimulating factor (M-CSF)/osteoprotegerin (OPG), mitogen-activated protein kinase (MAPK)s/c-Jun N-terminal kinase (JNK)/nuclear factor kappa-light-chain-enhancer of activated B cells (NF-κB), Kelch-like ECH-associated protein 1 (Keap-1)/nuclear factor erythroid 2–related factor 2 (Nrf2)/Heme Oxygenase-1 (HO-1), Bone Morphogenetic Protein 2 (BMP2)-Wnt/β-catenin, PhosphatidylInositol 3-Kinase (PI3K)/protein kinase B (Akt)/Glycogen Synthase Kinase 3 Beta (GSK3β), and other signaling pathways). Although majority of the studies on the osteoprotective properties of NPs against BDs were conducted *ex vivo* and mostly on animals, the use of NPs for treating human BDs and the prospects for future development remain promising.

## 1. Introduction

Bone diseases (BD) are characterized by inflammation, fractures, pains, and overall morbidity that hinder mobility and reduce the ability to indulge in various physical activities. These outcomes negatively impact the individual’s mental health conditions and/or those of their caretakers (often family members); as such, the need for the treatment of BDs cannot be overemphasized. The treatment of BDs involves the use of various medications and therapies such as antiresorptive and anabolic therapies. Most commonly used are bisphosphonates (e.g., alendronate, risedronate, ibandronate and zoledronic acid) [1] and selective estrogen receptor modulators (SERMs; e.g., tamoxifen, raloxifene, and toremifene) [2]. Although not completely understood, the mechanism of action of bisphosphonates is to inhibit the osteoclast-mediated bone resorption in a manner that is suggested to be influenced by the length of the R2 carbon chain, the presence of aromaticity, and the presence or absence of nitrogen in their structures [1,3]. In addition, bisphosphonates are applied in the treatment of Paget disease of the bones, cancers that have spread to the bones, multiple myeloma, and hypercalcemia of malignancy [4]. Bisphosphonates are frequently given for the prevention and treatment of a range of additional skeletal diseases, such as poor bone density and osteogenesis imperfecta, in addition to their already recognized applications [5]. However, numerous findings are linking the usage of bisphosphonates to pathologic diseases including fractures of the femur, depression and anxiety, inflammatory eye disease, and medication-related osteonecrosis of the jaw (MRONJ) [6,7,8]. MRONJ is a disease in which a portion of bone has stopped receiving blood flow. In patients with ONJ, sometimes referred to as avascular necrosis of the jaw, the jawbone becomes exposed. Frequently, the tooth that was above it comes out, leaving a painful, non-healing sore behind. The lower jaw suffers from osteonecrosis more frequently than the upper jaw, and this has increased scrutiny of the currently popular bisphosphonate treatment [9].

For SERMs, their mechanism of action is through the inhibition of estrogen activities achieved by their binding to and blocking the estrogen receptors [10]. For instance, zoledronic acid administered as a single intravenous dose inhibits the progression of Paget’s disease for approximately six years [1,11,12]. Both therapies are known to be effective in the treatment of various BDs, albeit, with various side effects and potential health concerns. While some side effects are mild, such as stomach upset and heartburn associated with bisphosphonate pills, some are more concerning, such as blood clots, stroke, and certain types of cancers associated with the SERMs [10]. In addition, anabolic steroids that are used in treating BDs are also linked to adverse effects which, in addition to those already mentioned for other drugs, include toxicity to vital organs such as the brain, liver, kidney, and reproductive organs, and impairment of cognitive and cardiovascular functions and several other systemic alterations [13,14]. Thus, the need arises for alternative treatments with little or no health concerns.

**Figure 1:**
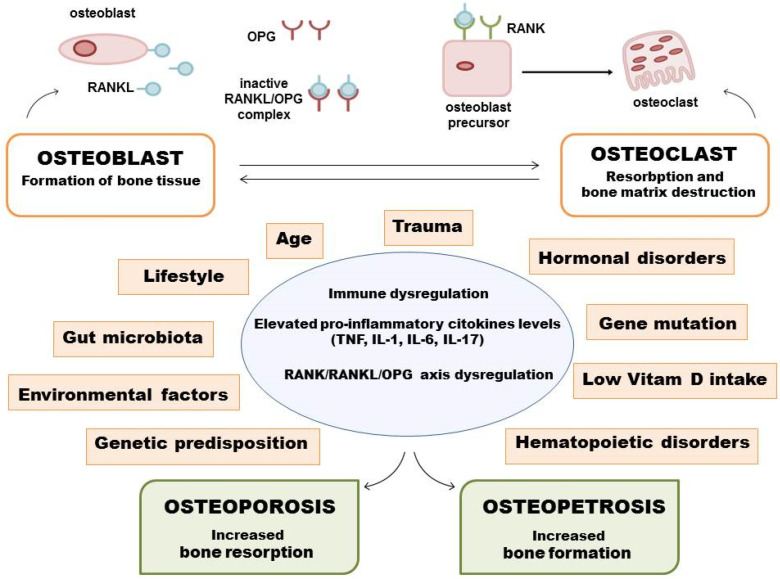
Risk factors for BDs.

Several factors such as age, trauma, hormonal disorders, mutations in osteoregulatory genes, deficiency in vitamin D, and others have been linked with BDs. These factors interact to increase someone’s likelihood of developing BDs [15]. For example, an individual with a mutation of *OPG,* the gene which codes for OPG (the protein that play significant roles in osteoprotection) has a higher chance of developing BDs at an earlier age, compared to individuals without such inherited mutation [16,17,18]. Similarly, older persons, especially postmenopausal women, have a higher risk of the disease due to estrogen deficiency [19]. Figure 1 illustrates risk factors for BDs.

NPs of plant origins such as fruits, onion, garlic, *Curcuma longa* L. (Zingiberaceae), and legumes have all been reported to elicit positive outcomes on bone health [20,21]. For instance, a 12-week study on ovariectomized (OVX) rats fed legume-added diets containing either soybeans, mung bean, cowpeas, or azuki beans reported that bone mass density (BMD) for spine and femur increased in all legume-fed rats when compared to the OVX rats fed non-legume containing diets (control) [22]. The authors also reported significant increases in the expression of RANKL and OPG with increases in the serum calcium and phosphate ratios for all OVX rats fed legume-enriched diets compared to the control [22]. In humans, the potential impacts of dietary proteins on the reduction of BDs was recently highlighted [23] and supported by two systematic reviews and meta-analyses studies conducted independently, which collectively reported that dietary protein intake improved femoral neck and total hip BMD, showed improvements on the lumbar spine, and significantly reduced cases of hip fractures [24,25]. In previous reviews, the pro-osteoanabolic and anti-osteocatabolic properties of extracts from different plant species were thoroughly discussed [26,27,28,29], motivating several new studies on the application of herbal materials in halting bone resorption in vitro and in vivo [30,31,32,33,34,35,36]. In this review, we aim to discuss the cellular and molecular mechanisms behind the health-promoting benefits of selected plant-derived and purely characterized NPs. Some health and safety concerns over the use of NPs are also briefly discussed, and our conclusions highlight some prospects for future studies. From reputable scientific databases and search engines such as PubMed, Google Scholar, and ScienceDirect, recent literature sources (2015–January 2022) on natural products with bone health-promoting effects and their mechanisms of action were retrieved. Search terms included “osteomodulatory”, “osteoanabolic”, “osteoprotective”, “natural products”, “bone health-promoting”, “bone health”, “phytochemicals”, and others. The abstracts were scanned for relevance, and then the whole articles were further read to include only papers discussing purely characterized natural products whose mechanisms of action in promoting bone health were investigated.

## 2. Pathological Mechanism of Development of Some Bone Diseases

Bone formation or modeling is achieved through a tightly regulated cascade of complex homeostatic cell–cell interactions between osteoclasts and osteoblasts. Osteoblasts are specialized cells of mesenchymal origin that are implicated in the synthesis of bone matrix and the regulation of bone mineralization [37]. Osteoblast activities starts after their differentiation from their parental mesenchymal lineage, a process mediated by RUNX2 (runt-related transcription factor 2) and osterix. RUNX2 and osterix are transcription factors whose absence in osterix-null mice and RUNX2-null mice lines lead to the non-production of osteoblasts or bones tissues, an indication that RUNX2 and osterix are central to the differentiation of osteoblasts [38,39]. One of the most important functions of bone is the hematopoiesis process, which takes place in the bone marrow [40]. Its homeostasis is maintained by a complex unit that mainly involves monocytes/macrophages, from which specialized cells called osteoclasts originate, which adhere to the bone matrix, thus causing the secretion of acid and lytic enzymes that degrade the bone matrix. The differentiation of osteoclasts from their parental lineage is achieved upon the stimulation of RANKL and the M-CSF [41]. The binding of M-CSF to the colony-stimulating factor-1 receptor (c-Fms) activates the distinct signaling pathways that ensure the survival of osteoclasts for the precursor and mature cells. However, RANKL has been identified as the primary osteoclast differentiation factor, as it regulates the expression of genes involved in osteoclast synthesis by binding to its receptor, known as RANK [41].

Collectively, it becomes apparent that the activities of osteoblasts and osteoclasts are antagonistic. Therefore, bone remodeling and quality depend on the tight regulation and maintenance of osteoblast–osteoclast activities in a homeostatic fashion. Alterations in the remodeling of bone can lead to various types of BDs, such as osteoporosis (OP), characterized by the shifting of the balance towards bone resorption at the expense of bone modeling [42]. OP is the most common BD and is characterized by low bone density (diagnosed as osteopenia), which leads to weak and brittle bones that are prone to fractures. Other common BDs are osteoarthritis (OA), which occurs due to the degeneration of cartilage tissues that causes bones between joints to rub together, resulting in inflammation, stiffness, and pain [43]. Rheumatoid arthritis (RA), as with OA, causes joint pains; however, while OA is due to wear-and-tear in the joints, RA is a chronic immunodeficiency disorder where the immune system attacks the linings of joint tissues. Gout is a joint-related BD that differs from OA and RA, in that it results from the accumulation of uric acid crystals in the joints (often due to the inability of the kidneys to process uric acid), which causes inflammation and pain [44]. Other BDs include Paget’s disease (bone deformation during remodeling), osteomyelitis (bone infection), osteonecrosis (bone death from blood starvation), bone tumors (uncontrolled cell growth within the bone), and scoliosis (abnormal curve in the spine bone) [45,46,47,48].

**Figure 2:**
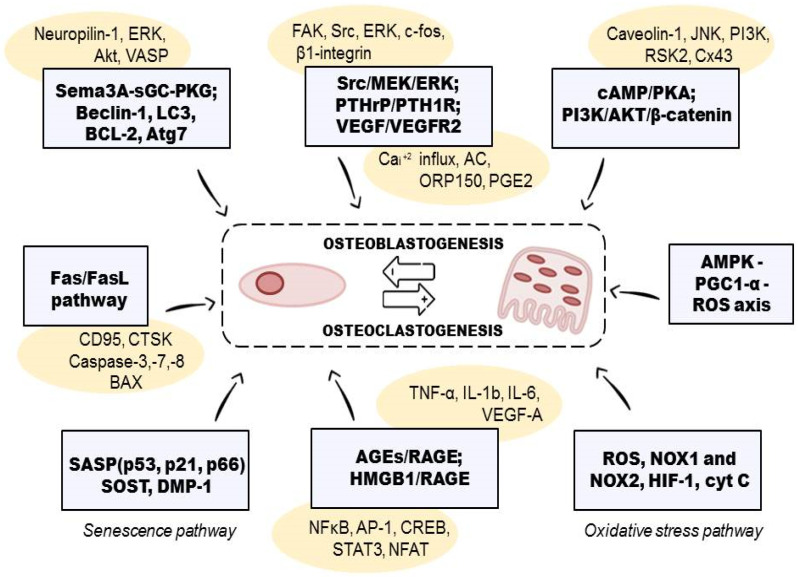
Molecular mechanism of bone resorption and the signaling pathways involved. SASP—senescence-associated secretory phenotype; SOST—sclerostin; DMP-1—dentin matrix acidic phosphoprotein 1; HIF-1—hypoxia-inducible factor 1; NOX-1/2—NADPH oxidase 1/2; cyt c—cytochrome C; ROS—reactive oxygen species; AGE/RAGE—advanced glycated end-products/receptor; HMGB1—high mobility group box 1; PTH—parathyroid hormone; AMPK—AMP-dependent kinase, PGC-1α—peroxisome proliferator-activated receptor gamma coactivator 1-alpha, tumor necrosis factor alpha; NF-ĸb—nuclear factor kappa B; TNF-α—tumor necrosis factor-alpha; p38 MAPK—mitogen-activated protein kinase; PGE2—prostaglandin E2, FasL—Fas ligand; IL-1b/6—interleukin 1 beta; IL-6—interleukin 6; VEGF-A—vascular endothelial growth factor alpha; CTSK—cathepsin K; IFN-γ—interferon gamma; CTR—calcitonin receptor, PI3K—phosphoinositide 3-kinase; Akt—protein kinase-B; GSK3β—glycogen synthase kinase-3β; JNK—c-Jun N-terminal kinase; ERK—extracellular signal-regulated kinase; tumor necrosis factor alpha; NF-ĸb—nuclear factor kappa B; TNF-α—tumor necrosis factor-alpha; p38 MAPK—mitogen-activated protein kinase.

With advancing age, the activity of osteoclasts tends to be greater than the activity of osteoblasts. In fact, physiological aging is characterized by a loss of bone mass, but the disease develops when this loss becomes excessive and pathological due to a persistent and dominant bone resorption activity compared to the new bone formation [49]. In recent years, it has emerged that the pathophysiological mechanisms that contribute to the onset of these diseases, in addition to the estrogen deficiency and the reduction of vitamin D intake that characterize postmenopausal osteoporosis, go way beyond this [50]. Cells of bone and of the immune system have many molecules in common, such as transcription factors, signaling factors, cytokines, or chemokines [51]; in particular, CD4^+^ cells stimulate the osteoclasts in the bone resorption process. A particular type of cell belonging to this group is T-helper 17 (Th17) cells, which derive from forkhead box P3+ (FOXP3+) T-cells, that under inflammatory conditions lose the expression of the transcription factor FOXP3, thus promoting the development of Th17 instead of regulatory T-lymphocytes [51]. Elevated levels of proinflammatory cytokines such as tumor necrosis factor (TNF), interleukin 1 (IL-1), interleukin 6 (IL-6), or interleukin 17 (IL-17) are also characteristic of osteoporosis [52] (Figure 2).

Other evidence of the involvement of the immune system is represented by B-cells, which regulate the RANK/RANKL/OPG axis, and which increase the production of RANKL in postmenopausal women [53,54]. Interestingly, new information has emerged on the interaction between homeostasis of bone metabolism and intestinal flora [55]. The set of microorganisms living in the human digestive tract influences the homeostasis of the gastrointestinal tract and the extra-gastrointestinal tract; in fact, the production and absorption of nutrients can be modulated. Germ-free mice have shown an increase in bone mass, highlighting a relationship between bone homeostasis and microbiota [56]. Moreover, the use of probiotics or antibiotics affects bone health [57]. Numerous treatments for osteoporosis have been developed, such as antiresorptive agents, while bone stimulators are all experimental and have not been approved by the Food and Drug Administration [58]. The general recommendation for all patients with osteoporosis is to ingest a physiological level of calcium and vitamin D, perform an appropriate exercise program, and adopt fall prevention.

A disequilibrium of bone turnover could lead to another skeletal dysplasia named osteopetrosis, a condition characterized by increased bone volume due to a dysfunction of the osteoclast activity or defects in the osteoclast formation. This condition produces deformities and structural fragility that is the cause of frequent fractures. In addition, affected patients have low blood cell production and loss of cranial nerve function, causing blindness, deafness, and facial nerve palsy [59]. According to the way in which the disease is inherited, it is classified into three different types: autosomal dominant, autosomal recessive, and X-linked recessive. The most common is the autosomal dominant form, which is characterized by mild symptoms that occur in late childhood through adulthood. The autosomal recessive form appears soon after birth and often shortens life expectancy. The X-linked form of osteopetrosis is extremely rare, with only a few reported cases [60]. The genes that cause the disease have been classified into the osteoclast-rich group and the osteoclast-poor group, where the latter includes mutations in RANKL and RANK genes [59,61]. A rare form of osteoclast-poor osteopetrosis depends on a mutation inside the RANKL gene that causes a deficiency of osteoclasts in bone tissue [62]; in particular, mutation on conserved residues alters RANKL homotrimerization with a potential impact on its function [63]. Given the nature of the disease, it may be useful to use genetic testing to confirm the diagnosis and to provide information on major organ involvement [64].

## 3. Cellular and Molecular Mechanisms of Bone Health-Promoting Properties of Some Natural Products

The maintenance of bone homoeostasis is dependent on the continuous removal of old and damaged bone tissues and the formation of new ones. These processes are linked to the activities of osteoclasts and osteoblasts, which are regulated by a cascade of interactions involving their respective signaling pathways [65,66]. This section presents the molecular mechanisms behind the actions of some NPs against bone malfunction initiated experimentally and their corresponding signaling pathways. As the major pathways associated with bone remodeling and pathogenesis of most bone diseases [42,67,68,69,70,71], the osteoprotective properties of NPs through the modulation of RANKL/M-CSF/OPG, MAPKs/JNK/NF-κB, Keap-1/Nrf2/HO-1, BMP2-Wnt/β-catenin, PI3K/Akt/GSK3β, and other signaling pathways are discussed in this section. Recently, the application of kaempferol, artemisinin, and its analogues as potential osteoprotective agents as demonstrated in various models of BDs has been succinctly reviewed [72,73]; consequently, these compounds were excluded from the present work.

### 3.1. Targeting the RANKL/M-CSF/OPG and MAPKs/JNK/NF-κB Signaling Pathways

Binding of RANKL and M-CSF to their respective receptors, RANK and c-Fms, initiates a network of biochemical events that induce osteoclastogenesis (OCG) and survival of osteoclasts [42]. OCG, which is the proliferation, differentiation, and maturation of osteoclasts, is an important event in the pathogenesis of bone resorption, OP, and other BDs [74]. These occur when the gene expression profile of OPG is downregulated [75]. Consequently, the molecules that inhibit both RANKL- and M-CSF-originated suppressions of OPG and also halt the clonal expansion of osteoclasts are the preferred candidates for osteolytic disease prevention and management [76]. Moreover, the induction of OCG by RANKL/M-CSF/OPG signaling occurs due to the upregulation of TNF receptor-associated factor-6 (TRAF6). TRAF6 activates a cascade of downstream events such as the extracellular signal-regulated kinase (ERK)/JNK/MAPK signaling, NF-ĸB/nuclear factor of activated T Cells 1 (NFATc1) signaling, Ca^2+^ signaling, and the PI3K/Akt/GSK3β signaling pathways, among others [77,78]. These signaling pathways lead to bone components’ degradation, whether through the CFos or Ca^2+^ signaling-mediated activation of the nuclear factor of NFATc-1, the transcription factor that upregulates the expression of several OC marker genes. Indeed, inflammation plays a significant role in OCG; thus, inflammation inducers such as lipopolysaccharides (LPS) are used in the experimental induction of OCG. LPS is a bacterial antigen that upregulates messenger ribonucleic acid (mRNA) expression of nuclear factor kappa B (NF-ĸB), which promotes the release of pro-inflammatory cytokines (TNF-α, IL-1β, and IL-6) and activates OCG [79,80]. Similarly, OCG induction can be achieved through hypercalcemia using chemicals such as 1α,25-dihydroxy-vitamin D3 [1α,25(OH)_2_D_3_] and prostaglandin E_2_ with a concomitant upregulation of RANKL [81].

The ongoing quest to identify NPs from diverse sources for the treatment and management of BDs has revealed prospects for herbs and fruits, which are rich in polyphenols and other phytochemicals [82]. For instance, the effects of alisol-B (**1**), a phytosteroid from *Alisma orientale* Juzepczuk, was investigated on 1α,25(OH)_2_D_3_-induced OCG in mouse bone marrow cells (BMMs) and primary osteoblasts [83]. The authors reported that alisol-B downregulated the level of phosphorylated c-Jun N-terminal kinase (JNK) and mRNA expression of NFATc1 and CFos, the major transcription factors needed for OCG and the consequent resorption of bones. Alisol-B 23-acetate was reported to block the mobilization of calcium ion (Ca^2+^), and its capability to reduce serum Ca^2+^ level and prevention of bone loss by over 50%, 95%, and 100% at 0.5, 1, and 5 µM, respectively, in a hypercalcemia mouse model after intragastric ingestion [84] indicates that alisol-B is biostable and bioavailable.

Desoxyrhapontigenin (**2**), a stilbene derived from *Rheum undulatum*, was shown to prevent OCG in RANKL-exposed BMMs by halting the activation of extracellular signal-regulated kinase (ERK) and suppressing the gene expression of c-Fos and NFATc1 [85]. In LPS-initiated bone loss in a mouse model, the stilbene also inhibited OCG by over 90% at 10 µM compared to 70% by resveratrol at the same concentration, and reduced bone resorption at 3, 10, and 30 µM by over 60%, 80%, and 95% by downregulating gene expression of matrix metalloproteinase-9 (MMP-9), tartrate-resistant acid phosphatase (TRAP), and cathepsin K (CTSK), which are target genes for NFATc1 and markers for OC abundance. Moreover, resveratrol activated the osteogenic transcription factor core-binding factor alpha 1 (Cbfa1) and NAD-dependent acetylase enzyme, Sirtuin 1 (Sirt-1), leading to the activation of differentiation and maturation of osteoblasts and osteogenesis. Furthermore, curcumin can reduce osteoarthritic environment-induced inflammation, extracellular matrix degradation, and chondrocyte apoptosis by targeting the NF-kB-Sox9 signaling axis [86]. In addition to the inhibition of RANKL-generated NFATc1, CTSK, and TRAP activation, another natural riboflavin-derived compound, lumichrome (**3**), suppresses calcium oscillation and activation of NF-κB and MAPKs needed for downstream events of the RANKL/M-CSF signaling pathway in RAW264.7 mouse macrophage cells (MMCs) and MC3T3-E1 pre-osteoblasts (MCPs) [87]. This anti-OCG property of lumichrome was confirmed in OVX mice, where the natural product inhibited bone loss by an approximately 2-fold decrease in osteoclast formation at 10 µM. Considering the role of estrogen in maintaining bone health, such as the promotion of type 1 collagen synthesis, osteoblast survival, and osteoclast apoptosis, and the inhibition of OCG [16,88], the depletion of estrogens in postmenopausal women is associated with several bone dysfunctions such as OP, osteoarthritis, and bone fractures [66,89]. Experimentally, estrogen deficiency is induced in female rodents by ovariectomy to mimic menopause-related estrogen depletion in aging women [90]. This animal model is widely adopted in the study of the potency of molecules for promoting bone health and preventing age-related bone degeneration [91].

Maslinic acid (**4**), a constituent of *Platostoma africanum* P. beauv., intraperitoneally-injected into OVX mice every 48 h for three months prevented loss of bone mineral density (BMD) [92]. It also elevated trabecular space/separation (TS) but reduced trabecular number (TN), OC number, and OC activities. In BMMs and MMCs, the pentacyclic triterpene acid also inhibited RANKL-induced OCG by over 3- and 5-fold, respectively, at 10 µM and actin ring formation by over 5-fold via blocking RANKL-induced activation of TRAF6, the transcription factor that enhances the expression of osteoclast marker genes (TRAP, MMP9, c-Src, CTR, and CTSK). Additionally, the molecular mechanism of anti-OCG activity of maslinic acid includes the inhibition of MAPKs and activation protein 1 (AP-1) activation and activating the nuclear factor of kappa light polypeptide gene enhancer in B-cells inhibitor (IκBα). The above findings suggest that phytochemicals such as resvertrol, berberine, maslinic acid, etc., can potentially target the RANKL/M-CSF/OPG and MAPKs/JNK/NF-κB signaling pathways to reduce the proliferation, differentiation, and maturation of OCs. Other natural compounds whose molecular mechanisms of anti-OCG and osteoprotective activities involve the modulation of RANKL/M-CSF/OPG and ERK/JNK/MAPK signaling pathways are presented in Table 1, such as an *Amaryllidaceae species*-derived alkaloid, lycorine (**5**) [93]; isoliquiritigenin (**6**) and astilbin (**7**), flavonoids isolated from *Glycyrrhiza glabra* L. [94] and *Astilbe thunbergii* (Siebold and Zucc.) Miq., respectively [95]; cimiracemate A (**8**), a *Cimicifuga racemose* L.-originated phenylpropanoid ester [96]; and a furanocoumarin isolated from *Notopterygium incisum* K.C.Ting ex H.T.Chang, notopterol (**9**) [97], among others.

### 3.2. Targeting Keap-1/Nrf2/HO-1 Signaling Pathway

**Figure 3:**
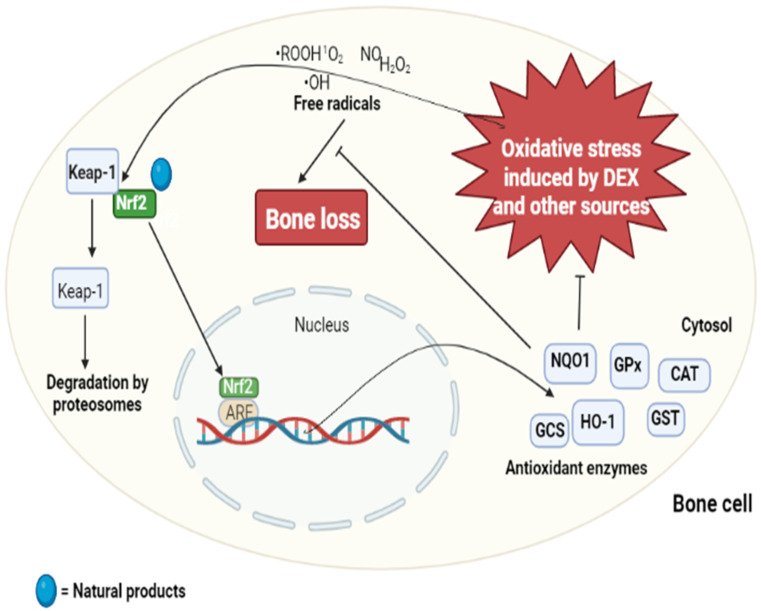
Modulation of bone remodeling by natural products via Keap-1/Nrf2 signaling pathway. Bone loss caused by oxidative stress due to an imbalance in the antioxidant and free radicals in the system can be alleviated by natural products with antioxidant ability. Natural products promote the cytosol-to-nuclear translocation of Nrf2 (when detached from its repressor, Keap-1, during increased ROS generation) to interact with its operator, ARE, to induce the expression of antioxidant enzymes. Increased availability of antioxidant enzyme will hence scavenge the ROS and prevent ROS-mediated bone loss. **Abbreviations**: GCS—γ-glutamylcysteine synthetase; HO-1—heme oxygenase-1; NQO1—NAD(P)H:quinone reductase; Keap-1—Kelch-like ECH-associated protein 1; Nrf2—nuclear factor E2-related factor 2; ARE—antioxidant response element; GPx—glutathione peroxidase; GST—glutathione-S-transferase; CAT—catalase; DEX—dexamethasone.

As intracellular signal molecules, reactive oxygen species (ROS) activate RANKL-mediated OCG and enhance the intracellular oxidative stress [116]. Hence, the reduction of ROS levels in bone tissue is a good target for osteoprotection [117]. When dissociated from its repressor, Kelch-like ECH-associated protein 1 (Keap-1), nuclear factor E2-related factor 2 (Nrf2) is translocated to the nucleus where its interaction with antioxidant response element (ARE) increases the gene expression for antioxidant enzymes that protect cells from ROS-induced cellular injury [118,119,120]. The enhanced expression of cytoprotective enzymes such as γ-glutamylcysteine synthetase (GCS), heme oxygenase-1 (HO-1), and NAD(P)H:quinone reductase (NQO1) as mediated by Nrf2 and the consequent suppression of OS has been shown to promote bone health [121,122]. In addition to the inhibition of OCG, other strategies to improve bone health involve protecting osteoblasts against externally and internally generated damages. In a recent investigation, a novel Keap-1 inhibitor, iKeap1, was shown to protect osteoblast from chemically generated oxidative assault and apoptosis by improving Nrf2-mediated expression of cytoprotective factors [123]. Treatment with a low dose of glucocorticoids, including dexamethasone (DEX), cortisone, and prednisolone, over a short time was shown to regulate bone remodeling and maintain homeostasis of the bone tissues [124]. However, administering the same drugs at a higher dosage over a long time elicited OP [125] and osteonecrosis via upregulation of glycogen synthase kinase-3β (GSK3β) gene expression and its enzyme activity [126]. Therefore, an overdose of DEX is helpful in the experimental induction of OP. Gastrodin (**30**), a phytochemical isolated from *Gastrodia elata*, was shown to protect murine osteoblastic cells (MC3T3-E1) from DEX-generated cellular assaults by improving cellular viability by 30% and the expression of osteogenic genes (RUNX2, osterix, bone morphogenetic protein-2 (BMP-2), and osteocalcin (OCN), and ALP activity [127], with over 0.15-, 0.3-, 2-, 0.2-, and 0.4-fold increases at 5 µM when compared to DEX-untreated group). In DEX-exposed rats, gastrodin prevented bone mineral loss, mitochondrial membrane dysfunction, and endoplasmic reticulum stress by suppressing apoptosis over 25% and 37.5% at 1 and 5 µM by reducing the levels of apoptosis-inducing factor, bax, cytochrome-C, and caspase-3 (by 50% at 5 µM compared to that DEX-induced untreated group), while activating Nrf2 signaling pathways [127]. The ability of gastrodin to offer protection against DEX-induced OP after oral ingestion is an indication of its bioavailability and biostability; these two important properties make it a suitable candidate for drug development towards OP prevention. Based on these findings, natural products such as gastrodin, icariin, and chlorogenic acid with antioxidant ability via the binding to and translocation of Nrf to the nucleus to upregulate the expression of antioxidant molecules will alleviate ROS-mediated bone loss. Besides gastrodin, there are other NPs such as icariin (**31**) [128], chlorogenic acid (**32**) [129], and 4-phenyl butyric acid (**33**) [130] that attenuate DEX-induced alteration in structure and function of bone tissues, potentially via modulation of the Keap-1/Nrf2/HO-1 signaling pathway (Figure 3).

### 3.3. Targeting BMP2-Wnt/β-Catenin Signaling Pathway

RUNX2 is a human protein that regulates the gene expression of the extracellular matrix protein encoded by the RUNX2 gene. The gene of RUNX2 protein is expressed early in the mesenchyme of an embryo; RUNX2 modulates the generation of skeletal components before the bone tissues are formed. The BMP-2/Wnt/β-catenin signaling pathway regulates RUNX2 activity [87]; extracellular bone morphogenetic protein-2 (BMP-2) activate serine-threonine kinase receptors I and II, causing the phosphorylation and translocation of SMAD (SMA, “small” worm phenotype, and MAD, “mothers against decapentaplegic”) proteins into the nucleus to upregulate the expression of osteogenic genes such as RUNX2 [131] and osteocalcin [132]. Similarly, BMP activates the movement of β-catenin into the nucleus to initiate osteoblast development via the canonical Wnt/β-catenin signaling pathway that elevates osteoblastogenesis (OBG) and bone production [133,134]. The secreted Wnt proteins bind to the frizzled receptor and co-receptors (LRP5/6) to inhibit adenomatosis polyposis coli (Apc)-GSK3β-actin complex and prevent the phosphorylation-mediated breakdown of β-catenin by the proteasome. Therefore, β-catenin builds up in the cytosol, and is transported into the nucleus where it binds to its promoter regions, TCF and LEF, to stimulate the transcription of target genes such as the RUNX2 gene, C-myc, cyclin D1, and c-jun. The nuclear activity of β-catenin includes the expression of osteogenic proteins such as osteocalcin (OCN), alkaline phosphatase (ALP), and bone sialoprotein (BSP) [134,135]. When in a dephosphorylated state, its breakdown in proteosomes is induced by active GSK3β phosphorylates β-catenin, which leads to a reduction in its cytosolic level [136,137]. Hence, GSK3β is a regulator of BMP-2/Wnt/β-catenin signaling pathways at the Wnt/β-catenin axis.

The most prevalent therapeutic strategies for treating OP include the administration of estrogens, bisphosphonates, vitamin D analogues, and RANKL inhibitors [138]. Although effective in the management of OP, these drugs increase the risk of obesity and diabetes and metabolic syndrome, in general [139,140]. Consequently, NPs with little or no side effects are of interest in the research on the prevention and treatment of OP and related diseases. Low-molecular-weight compounds of natural origin have been reported to boost osteoblastogenesis (OBG) in several *in vitro* studies [141,142], including those that act via Wnt/β-catenin signaling pathway [137,143]. Notably, albiflorin (**34**) and paeoniflorin (**35**) isolated from *Paeonia lactiflora* enhanced OBG in MC3T3-E1 cells by upregulating the mRNA expression of Wnt (Wnt10b), β-catenin (ctnnb1), LRP5, LRP6, Dvl2, and cyclin D1 (Ccnd1) [144], suggesting the involvement of BMP2-Wnt/β-catenin signaling pathway. Other NPs that promote bone health either by inhibiting OCG or promoting OBG and fracture healing via BMP2-Wnt/β-catenin signaling pathway include quercetin (**36**), a flavonoid widely found in fruits and vegetables [145]; polydatin (**37**), a precursor of resveratrol [134,146,147]; and 3,5-dicaffeoyl-epi-quinic acid (**38**) from *Atriplex gmelinii* [140]. In general, NPs such as albiflorin and paeoniflorin with the ability to target BMP-2/Wnt/β-catenin signaling pathway to upregulate the RUNX2 gene required for bone health are potential agents to ameliorate BDs such as OP.

### 3.4. Targeting PI3K/Akt/GSK3β Signaling Pathway

The phosphoinositide 3-kinase/protein kinase-B (PI3K/Akt) signaling pathway is well known due to its multifunctionality in biological processes, including maintenance of glucose and bone homeostasis [148]. Studies on cell proliferation and death have always focused on the PI3K/Akt/GSK-3β signaling pathway and the BMP-2/Wnt/β-catenin signaling pathway [149]. The PI3K, an intracellular phosphatidylinositol kinase with several catalytic subunits, is activated by the interaction between an extracellular signal molecule and activated Ras protein receptor, leading to the phosphorylation of PIP2 to PIP3, which activates Akt by phosphorylation [150]. The active Akt, in turn, phosphorylates the ser-9 of GSK3β and inactivates it, making β-catenin available to play roles in the upregulation of expression of genes that code for osteogenic proteins [151]. DEX has been shown to elevate ROS production, which can lead to apoptosis through the mitochondrial caspase apoptosis pathway [152]. Furthermore, the accumulation of ROS causes oxidative stress in osteoblasts, activating the JNK pathway, which inactivates the Akt pathway and promotes osteoblast death [153]. Vinpocetin (**39**), a small chemical derived from the leaves of *Phyllostachys pubescens*, attenuates DEX-induced rat osteoblast apoptosis by 60% and 65% at 5 and 10 µM than that caused by DEX and inhibits the progression of osteonecrosis of the femoral head (*ONFH*) by lowering DEX-induced elevated levels of ROS by over 90% at 10 µM and activating the Akt pathway via Akt phosphorylation by over 2-fold at 10 µM. Vinpocetin had no effect on osteoclast differentiation and prevented DEX-stimulated protein upregulation, including cleaved-caspase3 and Bax, while dramatically suppressing Bcl-xl and Bcl2 downregulation [154] by over 2-fold each.

Epigallocatechin gallate (EGCG) (**40**) is one of the best known catechins of green tea due to its diverse biological activities, including the prevention and treatment of diseases [155]. EGCG initiates apoptosis of RAW 264.7 cells, differentiated OCs. by 10-fold at 50 µM when compared to control through the stimulation of caspase, thereby preventing alveolar bone resorption while promoting osteogenesis [156,157]. It also enhances mineralization of human osteoblast-like cells [157,158], the proliferation and differentiation of successfully passaged human alveolar osteoblasts (hAOBs) and human periodontal ligament cells (hPDLCs), and prevented bone loss by significantly and dose-dependently increasing mRNA expressions of ALP, RUNX2, BMP2, OSX, and OCN by 2-, 7-, 6-, 5-, and 4-fold, respectively, at 10 µM concentrations via the regulation of PI3K/Akt signaling pathway [157]. Imperatorin (**41**), a linear furocoumarin derived from remarkable components of traditional Chinese medicine, *Angelica archangelica* and *Peucedanum praeruptorum*, enhanced OBG in rat BMMs at 75 µM via activation of RUNX2, COL1A1, and osteocalcin by 5-fold each and by promoting the phosphorylation of Ser473 of Akt. This is followed by an enhanced phosphorylation of Ser9 of GSK3β, and suppression of OCG via the Akt/GSK3β/β-catenin signaling pathway [151]. Therefore, NPs such as vinpocetine scavenge ROS by inhibiting the JNK pathway and at the same time activating the PI3K/Akt/GSK-3β signaling pathway, leading to the expression of osteogenic genes as a suitable therapy for BDs. Other natural compounds that boost bone health by reducing OCG or enhancing OBG and fracture-healing via Akt/GSK3β/β-catenin signaling pathway include sesamin (**42**), a lignan isolated from *Sesamum indicum* [152]; garcinol (**43**), a polyisoprenylated benzophenone extracted from the fruit of *Garcinia indica* [159]; and others listed in Table 2.

### 3.5. Targeting Rev-Erbs Signaling Pathway

The nuclear receptors, Rev-erbs, consisting of Rev-erbα and Rev-erbβ, corresponding to nuclear receptor subfamily 1, group D, members (NR1D) 1 and 2, play multiple physiological roles, such as the regulation of the circadian rhythm, bone health, and inflammatory processes [162,163]. Circadian clocks have been shown to regulate bone remodeling through the controlling of formation and resorption of bones [164]. The role of Rev-erbs in maintaining bone homeostasis has been demonstrated in knockout mouse where deletion of Rev-erbα gene increased inflammation, mitochondrial dysfunction, and oxidative stress, and promoted OCG [163]. However, the activation of Rev-erbβ inhibits OCG and prevented chemical-induced loss in bone mass [165,166]. Specifically, the activation of Rev-erbα prevents RANKL-generated podosome belt synthesis and prevents osteoclast-induced bone resorption, thereby suppressing OVX-induced bone loss. In addition, the activation of Rev-erbα, using its agonists SR9009 and GSK4112, abrogates OCG, as characterized by the suppression of NF-κB, NFATc1, and CFos and their downstream effectors, TRAP, MMP9, and CTSK [165], and inhibited LPS-generated inflammation [167]. Therefore, Rev-erbα exerts a crucial role in maintaining healthy bone status by inhibiting OCG while promoting osteogenesis. NPs have been shown to alleviate oxidative stress-induced diseases, including OP, and metabolic disorders by regulating the Rev-erbα/miR-882 pathway. For instance, berberine (**45**), isolated from *Rhizoma coptidis*, was reported to be an agonist of REV-ERBα in a colitis model [168]. Nevertheless, the effects of berberine on Rev-erbα are both dose- and “time-of-the-day”-dependent [168,169]. Thus, a comprehensive understanding of the impacts of berberine and Rev-erbs interaction in relation to bone health will require further studies in the future. It would also be interesting in the future to examine the osteoprotective effects of natural modulators of Rev-erbs expression and activities. In sum, bone health is improved with natural products such as berberine to alleviate chemical and OVX bone loss and RANKL-generated and osteoclast-induced bone resorption via the modulation of the Rev-erb signaling pathway.

### 3.6. Targeting Calcium Ion Signaling Pathway

Calcium is one of the major and ubiquitous components of bone found mostly in the form of calcium hydroxyapatite (Ca_10_[PO_4_]_6_[OH]_2_) [170]. Calcium, to a very large extent, contributes to the homeostasis and regulation of bone metabolism and bone resorption via many elucidated and yet-to-be-unraveled pathways. Moreover, calcium forms one major regulatory component of the osteoblast and osteoclast and plays a role in determining the fate of mesenchymal cells in humans [171]. Several hormones, cytokines, and matrix-embedded factors play invaluable roles in the maintenance of the balance between extracellular calcium and intracellular calcium [172]. Calcium-sensing receptors (CaSR) are vital parathyroid, seven-transmembrane G-protein couple receptors, which are also present in the kidney, and function in the maintenance of homeostasis of bone by controlling the influx and release of extracellular calcium from bone cells [173]. An increase in Ca^2+^ activates CaSR, which causes an influx of Ca^2+^ into the osteoblast, thereby promoting bone remodeling. However, when there is a decrease in extracellular Ca^2+^, CaSR causes resorption of Ca^2+^ from distal tubules of the kidney as well as fostering bone resorption by osteoclasts [174]. When the extracellular Ca^2+^ is increased, maybe as a result of increased osteoclastic activities, CaSR stimulates the influx of Ca^2+^ into osteoblasts by activating phospholipases C, A_2_, and D [172].

Primarily, phospholipase C fosters the formation of the second messenger (diacylglycerol (DAG) and inositol-1,4,5-trisphosphate (IP3)) from the cleavage of phosphatidylinositol 4,5-bisphosphate (PIP2) as well as the activation of phospholipases A2 and D, whose role in maintaining bone balance is poorly understood [175]. While the DAG remains membrane-bounded, IP_3_, which is soluble, diffuses out of the parathyroid cells and binds to calcium channel receptors (inositol trisphosphate receptor—IP3R) located in the endoplasmic reticulum, thereby fostering the release of Ca^2+^ in the cytosol and activating other Ca^2+^-regulated signaling axes [175]. Alternatively, intracellular calcium, especially in osteoblast cells, exerts some form of regulation on the bone homeostasis process. Hormones such as vitamin D3, insulin-like growth factor (IGF), and parathyroid hormone (PTH), including several other cytokines and growth factors, modulate the bone remodeling process mainly by influencing the amount of intracellular calcium in the osteoblasts [174]. Most of these hormones facilitate the translocation of extracellular calcium into intracellular osteoblast cells via the L-type and non-L-type isoforms of voltage-gated calcium channels. Increases in intracellular Ca^2+^ concentration consequently activate the CaMK-dependent pathway, which fosters an increased rate of cell entry to cell cycle and osteoblast proliferation [176]. Like the influence of calcium on extracellular CaSR-mediated and intracellular endocrine-mediated bone homeostasis, natural compounds and their synthetic counterparts act as CaSR agonists (calcimetics) or antagonists (calcilytics), and as such, regulate bone resorption-modeling balance [177]. This-subsection focuses on NPs that can improve bone health through the Ca^2+^ signaling axis. Several studies have identified some phytochemicals and bioactive peptides to play vital roles in maintaining bone health through the regulation of CaSR activities and other Ca^2+^ signaling pathway. Feng et al. [174] identified ligustroflavone (**47**) from *Ligustrum lucidum* with the ability to inhibit CaSR and improve the level of parathyroid hormones in HEK-293 cells and the serum of diabetic mice, respectively. In contrast, a similar study reported an upregulation of CaSR in human aortic endothelial cells administered with a dipeptide, γ-Glutamyl valine (γ-EV), from edible beans [173]. Although the report was directed towards the management of diabetes and intestinal inflammation, the CaSR mRNA, which showed suppression in one study and upregulation in the other, also plays a vital role in the CaSR axis of bone homeostasis [173,174]. In conclusion, NPs that modulate CaSR activities and other calcium signaling pathway could regulate OCs, osteoblasts, and extracellular calcium ion balance, resulting in bone remodeling.

Several other studies, as summarized in Table 3, have investigated some other natural products on other axes of Ca^2+^ signaling, such as on type 1 collagen [178], calmodulin phosphor kinase II [176], L-type Ca^2+^-gated channel [179,180], and myosin light chain kinase [181]. There is a need for more focused studies on the management and improvement of bone health through the several Ca^2+^ signaling axes.

### 3.7. Targeting Endogenous Molecules with Bone Promoting Properties

In addition to natural compounds sourced externally, targeting the upregulation of endogenous molecules can be an additive strategy for promoting bone health. For example, an extracellular matrix protein, ameloblastin (51), inhibited RANKL/M-CSF-induced OCG in MMCs by suppressing NFATc1 activation via inhibition of p38- and JNK-mediated upregulation of CFos [183]. Further supporting this report on irisin, an endogenous molecule from human dental bud was shown to promote bone formation in cultured MMCs [184]. Similarly, an endogenous methoxyindole, melatonin (N-acetyl-5-methoxy-tryptamine) (52), was recently shown to halt OCG in MMCs exposed to RANKL and M-CSF by downregulating MRNA expression of CTSK and its protein level via the upregulation of expression of nuclear receptor subfamily-1 group D member-1 (NR1D1, also known as Rev-erbα) with a concomitant downregulation of miR-882 [185]. An in-depth exploration of melatonin towards improving bone health using preclinical and clinical studies has been given elsewhere [186]. Other natural products such as maackiain, cycloastragenol, gamabufotalin, abrine, hymenialdisine, oroxylin A, demethylbelamcandaquinone B, and pitavastatin have been recently shown to promote bone health by enhancing the differentiation and maturation of endogenous molecules that inhibit bone resorption and/or promote osteoblastogenesis, and some of the compounds such as hymenialdisine, cycloastragenol, 12-deoxyphorbol 13-acetate, aerophobin-1, and pitavastatin have been demonstrated to be bioactive in both in vitro and in vivo experiments [187,188,189,190,191,192,193,194,195,196]. Nonetheless, further studies towards the enhancement of the protein levels of these endogenous osteoprotective molecules and their receptors hold promise towards finding novel interventions in the prevention, treatment, or management of BDs. Based on the above findings, compounds such as ameloblastin, abrine, oroxylin, and pitavastatin can potentially promote bone health by targeting the upregulation of endogenous molecules that can inhibit RANKL/M-CSF-induced OCG.

### 3.8. Targeting Vitamin D Receptor (VDR)

In an OPG-deficient murine model, intragastric ingestion of 1α,25-dihydroxyvitamin D_3_ [1α,25(OH)_2_D_3_] was shown to halt bone resorption and erosion, despite the elevated activation of RANKL/RANK signaling pathway [81]. However, 1α,25(OH)_2_D_3_ prevented M-CSF- and RANKL-induced OCG in cultured MMCs via suppression of gene expression of CFos protein [81]. This implies that the activation of VDR abrogates OCG by preventing CFos-mediated activation of NFATc1 and that vitamin D and its analogues exert their osteoprotective properties by targeting CFos-induced OCG. EGCG (40) was shown to exhibit an anti-OCG property by preventing IL-1-induced bone loss via targeting the gene expression of CFos and its protein activation [197]. However, whether VDR is involved in the downregulation of CFos gene expression and the suppression of its capacity to induce OCG and bone loss by EGCG remains to be investigated. In sum, NPs’ bone remodeling capacity occurs by activating VDR suppression of CFos protein expression, leading to the reduction in OCs.

**Figure 4:**
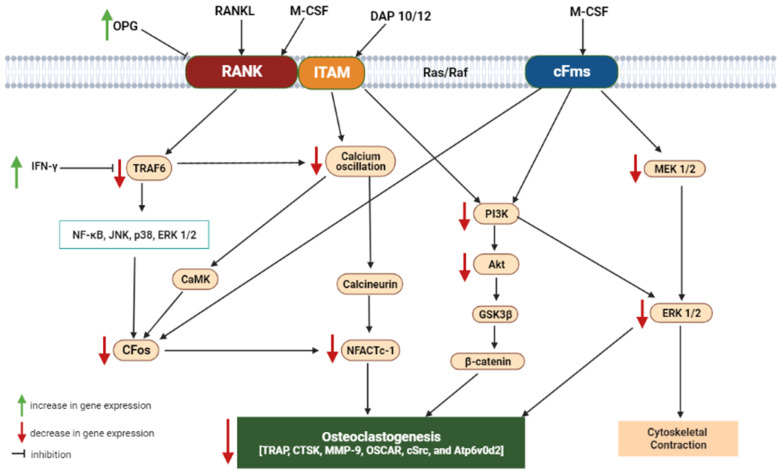
Summary of signaling pathways through which natural products exert their osteoprotective properties, in addition to the Keap-1/Nrf2 signaling pathway. OPG—osteoprotegerin; NFATc-1—nuclear factor of activated T-cells cytoplasmic 1; CaMKII—Ca^2+^/calmodulin (CaM)-dependent protein kinases; IFN-γ—interferon gamma; AP-1—activator protein 1; ATP6v0d2—ATPase Hþ transporting V0 subunit d2; CTR—calcitonin receptor; OSCAR—osteoclast-associated receptor; TRAF6—tumor necrosis factor (TNF) receptor-associated factor-6; TRAP—tartrate-resistant acid phosphatase; RANKL—receptor activation of NF-κB ligand; M-CSF—monocyte/macrophage colony stimulating factor; MMP-9—matrix metalloproteinase-9; CTSK—cathepsin K; PI3K—phosphoinositide 3-kinase; Akt—protein kinase-B; GSK3β—glycogen synthase kinase-3β; JNK—c-Jun N-terminal kinase; ERK—extracellular signal-regulated kinase; tumor necrosis factor alpha; NF-ĸb—nuclear factor kappa B; TNF-α—tumor necrosis factor-alpha; p38 MAPK—mitogen-activated protein kinase.

Figure 4 summarizes the major pathways by which NPs inhibit OCG and the associated bone loss, in addition to the Keap-1/Nrf2 signaling pathway, while Figure S1 (in Supplementary Materials) presents the structures of NPs scientifically demonstrated to have beneficial effects on bone health.

## 4. Clinical Trials of Natural Products for Bone Diseases

Despite the numerous interesting outcomes on the efficacy of natural products on bone health as reported from a variety of *in vitro* cell lines and *in vivo* animal studies, the findings cannot be validated as efficacious in humans without proper clinical studies. A few clinical studies have shown that some natural products from plants and animals have improved the health condition of osteoarthritis and osteopenia patients, especially in the postmenopausal population (Table 4). Most of the studies adopted randomized, parallel interventional, double-blinded, placebo-controlled models for the trials. Generally, changes in certain bone parameters such as the increase or maintenance of BMD and bone formation marker proteins (N-terminal propeptide of type I collagen (P1NP), collagen type 1 cross-linked C-telopeptide, and bone-specific alkaline phosphatase (BSAP)) are important indicators of bone health improvement in patients with unhealthy bone conditions. Conversely, an increase in bone resorption markers (such as osteocalcin, carbo-terminal telopeptide of type I collagen (CTx), and TRAP) and a concomitant decrease in BMD are indications of osteopenia/osteoarthritis progression (Table 4).

In a study by Shen et al. [198], polyphenols from green tea administered as an oral formulation daily to 171 osteopenic postmenopausal women successfully led to a significant increase in the bone formation marker BSAP and a decrease in the bone resorption factors TRAP in a ratio of 103.6% when compared to the baseline (100%) [198]. Another study reported a similar increase in the BMD of L2-L4 lumbar spine vertebra (*p* < 0.05), femoral neck (*p* < 0.01), and trochanter (*p* < 0.01) of the treatment group administered 80 mg/d of isoflavone aglycones-rich red clover extract supplemented with calcium (1040 mg/d), vitamin D (25 µg/d), and magnesium (487 mg/d), when compared to the placebo control group administered only the supplement [202]. A similar outcome was obtained with hop rho iso-alpha acids (200 mg) and berberine sulphate trihydrate (100 mg) [200,201], collagen peptide from pork skin and bovine bone [203,205], prenylflavonoids from *Epimedium* spp. [204], kefir-fermented milk peptides [206], and dried plum [207].

There is a need for more clinical studies on other natural products, especially from plant phytochemicals and bioactive peptides, to ensure that they are not just efficacious but also non-toxic (without adverse effects) to humans. Only a few clinical studies have investigated the adverse effects of natural products alongside their effects on bone health [204]. Moreover, most of the available clinical studies investigated the effects of natural products on bone by administering formulations orally to the volunteered human subjects; there are very sparse or no available studies that have administered their natural formulation through intramuscular or intravenous in human subjects. There is a possibility that better bone regeneration and improvement effects could be achieved through intramuscular or intravenous injection, as suggested from the results of *in vivo* animal studies (Table 4). 

## 5. Safety Concerns of Applying Some Natural Products for Clinical Management of Bone Diseases and Limitations of Some of the Current Studies

Although many NPs possess bone health-promoting properties, such as the prevention of bone resorption via anti-OCG or the improvement of the renewal of new bone cells and its protectants via OBG and upregulation of OPG expressions, the potential of some NPs to induce OCG should be approached with caution. For instance, intragastric injection of psoralen, a derived compound from *Psoralea corylifolia* L., when administered at 20 mg/kg for four weeks was reported to promote fracture healing in a rat transverse tibial fracture model. In contrast, the same compound (psoralen) was reported to enhance rat bone resorption and OCG in MCPs and BMMs via the activation of the ERK signaling pathway [208]. In molecular biology, psoralen is utilized as a mutagen. Psoralen plus ultra violet radiation (UVA) (PUVA) therapy is a combination of psoralen and UVA that can be used to treat hyperproliferative skin conditions including psoriasis and some types of skin cancer [209]. Unfortunately, the use of PUVA increases the risk of skin cancer [210]. Similarly, a recent study demonstrated that puerarin isolated from *Puerariae radix* alleviates hyperhomocycteinemia by antagonizing Rev-erbα, a receptor whose activation is known to protect the bones [211]. These findings confounded the roles of psoralen and puerarin on bone health; thus, they would not be suitable candidates for drug development in relation to fracture and hyperhomocycteinemia management. Some of the studies reviewed also have limitations in terms of the clinical usefulness of the study design. For example, a study by Jinhua et al. [128] reported that the administration of icariin at 250 mg/kg/d for 60 days protected against DEX-induced OP in mice. However, at lower doses (10 and 20 mg/kg/d), Jang et al. [98] reported that the administration of protocatechuic acid for 12 weeks protected against trabecular bone loss in OVX mice. The study designs of the above researchers as well as their findings are not translatable in clinical practice due to either remarkably high doses that will create safety concerns and/or very long duration of treatment, making these NPs competitively low compared to current drugs for managing bone diseases. Future researchers should adopt study designs that are clinically feasible, such as administering low doses and shorter treatment duration. For instance, at 1 and 5 mg/kg for 7 days and at 5 and 10 mg/kg/d for 7 days, garcinol and sarsasapogenin, respectively, were reported to attenuate LPS-induced calvarial osteolysis mice [99,159]. The design of the above studies is translatable into clinical practice with minimal concern for drug adherence and toxicity.

Some natural bioactive chemicals are produced by plants and marine organisms to protect them against predators due to their poisonous nature [212,213]. With the growing interest in medicinal plants, there is a need for more rigorous scientific research into their usefulness and toxicity. Natural products, e.g., lectins such as concanvalin A, found in plants such as cereal and beans were discovered to have toxic effects, including disrupting digestion and causing nutrient deficiencies; they can also elicit IgG and IgM antibodies, leading to food allergies [214,215]. These compounds can bind to erythrocytes simultaneously with immune factors, causing hemagglutination and anemia. In experimental animals, lectins impair host resistance to infection, induce failure to flourish, and even cause death [216]. In addition to organ toxicities [217] and gastrointestinal tract discomfort [218], a number of these plants and their chemicals induce genotoxicity, which could result in carcinogenesis [219]. Similarly, a marine glycoprotein known as shrimp tropomyosin was demonstrated to induce serious allergic reaction in an animal model, which can be abolished by glycation [220] It is therefore important to be cautious in selecting only natural products which are validated to be safe and patients are not allergic to, as the consumption of natural products whose safety is not substantiated could pose risks to human health. Efforts should be made to certify both the short- and long-term safety profile of NPs before their administration as drugs.

## 6. Conclusions and Prospects

Bones disease is a global health concern, and as such, there is an ongoing quest to identify the most effective treatments with little or no side effects. Current medications for treatments cause side effects that lead to more serious health conditions such as cancer. Thus, the search for alternative treatments has revealed the efficacy of NPs, many of which are of plant origins known to exhibit little or no side effects; moreover, some of these, such as soybeans, are part of the human diet. Thus, natural products’ preventive benefits in bone diseases such as osteoporosis might be a viable alternative, or at least serve as complementary therapeutics to conventional therapy to minimize side effects. However, the effects of combining NPs and conventional drugs on pharmacokinetics and their safety profile need to be clearly-established prior to adoption. In this review, we described the mechanisms behind the actions of some NPs towards the treatment of BDs. However, these are not exhaustive. There is need for future studies to focus on discovering more NPs and their corresponding mechanisms of action. Moreover, many of the studies on BDs have been conducted in other animals such as mice and rats; thus, it would be a welcome development if human studies on bone could be improved, especially studies using plants that are part of the human diet. The concern over dosage has remained one of the hinderances in the wider adoption of NPs in relation to the treatment of diseases. Although this is valid and perhaps justifiable, more emphasis should be laid on the consumption of diets rich in these NPs, especially for BD patients and those prone to developing such diseases.

Indeed, many of the NPs with potential to halt, reduce, or treat bones diseases originate from plants, but they are produced in small quantities, which hinders their adoption on a commercial level. However, plant breeding and the relevant technologies have improved over the years, such that the production of various phytochemicals can be manipulated through multiple strategies. Therefore, future research studies on NPs of plant origin in relation to bone diseases treatment should incorporate some plant breeding techniques with potential to improve the accumulation of the products of interest. Taken together, this review illustrates that the future of NPs in the treatment of BDs is promising but achieving this will rely on future research.

## Figures and Tables

**Table 1:** Natural compounds with osteoprotective properties via RANKL/M-CSF/OPG and MAPK/JNK/NF-ĸB signaling pathways.

Natural Compound	Source	Study Model	Specific Therapeutic Activity	Ref.
**Alisol-B (1)**	*Alisma orientale* Juzepczuk	1α,25(OH)_2_D_3_-induced OCG in BMMs and primary osteoblasts	Prevented bone loss (50–100%) by downregulating the expression of pJNK, NFATc1, and CFos. Reduced serum Ca^2+^ level at 0.5–5 μM.	[84]
**Desoxyrhapontigenin (2)**	*Rheum undulatum*	RANKL-exposed BMMs	At 10 μM, it inhibited OCG by 90% compared to resveratrol (70%) and reduced bone resorption (60–95%) at 3–30 μM by downregulating the expression of MMP-9, TRAP, CTSK, and NFATc1.	[85]
**Lumichrome (3)**	**Milk**	OVX mice	Inhibited bone loss by 2-fold at 10 μM.	[87]
**Maslinic acid (4)**	*Platostoma africanum* P. beauv.	OVX mice	Prevented loss of bone mineral density (BMD).	[92]
		RANKL-induced OCG in BMMs and MMCs	At 10 μM, inhibited RANKL-induced OCG (3- and 5-fold), MAPK, actin ring formation (5-fold) by blocking activation of AP-1, TRAF6, TRAP, MM9, s-Src, CTR, and CTSK, and activation of IκBα.	
**Lycorine (5)**	***Amaryllidaceae* SPP.**	RANKL-induced OCG in BMMs	Inhibited RANKL-induced OCs expression and bone resorption by 70–99% at 0.1–0.4 μM, thereby suppressing the expression of TRAP (70%), CTR (67%), NFATc1 (33%), CTR (60%), DC-STAMP (75%), and CTSK (90%) at 0.4 μM at day 5.	[93]
		**OVX mice**	Prevented OVX-induced bone loss and titanium particle-induced osteolysis reducing expression level of TRAP, V-ATPase, NFATc1, CTR, and CTSK between 25% and 90% at 0.4 μM compared to control, but increased TN, TT, and BV/TV levels by at least 20% at 0.5–2.5 mg/kg.	
**Isoliquiritigenin (6)**	*Glycyrrhiza glabra* L.	RANKL-induced OCG in BMMs and RAW 264.7 cells	Inhibited OC differentiation in both cells. At 20 μM, reduced bone resorption (95%), gene expression of NFATc1, C-Fos, TRAP, CatK, and MMP-9 between 50% and 98%.	[94]
		C57/BL6 mice	Prevented LPS-induced bone erosion in mice by 65% and reduced OCs formation by 55%.	
Astilbin (7)	*Astilbe thunbergii* (Siebold and Zucc.) Miq.	OVX mouse	Reduced OCs and TS levels by 10-fold while increasing the expression of BVD and TN by over 50% at 10 mg/kg for 6 weeks.	[95]
		RANKL-induced OCG in BMMs	Decreased expression of NFATc1, CFos, ACP5, CTSK, NF-κB, JNK, ERK, and p38, and Ca^2+^ oscillation by 50%, 50%, 50%, 70%, 70%, 50%, and 30%, respectively, while increasing IκBα by 1% at 10 μmol/L in cell culture after five days treatment.	
Notopterol (**9**)	*Notopterygium incisum* K.C.Ting ex H.T.Chang	OVX mice	Inhibited bone loss by reducing F-actin ring formation and resorption indices by 50% at 10 mg/kg for 6 weeks.	[97]
		RANKL-induced OCG in BMMs and MMCs	The gene expression profiles of CTSK, ACP5, V-ATPase-d2, JNK, ERK, NFATc1, CFos, TRAP, CTR, MMP-9, NF-kB, and TRAF6 and ROS production were decreased by between 30% and 75% while the expressions of IκBα, GSR, CAT, NQO1, Nrf2/Keap1, and ARE were increased by 25%, 55%, 50%, 30%, 33%, and 300%, respectively, at 10 μM after 5 days treatment.	
Protocatechuic acid (**10**)	*Rubus coreanus* Miquel	OVX mouse	At 20 mg/kg/d, protocatechuic acid attenuated OVX-induced bone loss by reducing gene expression profiles of RANKL, ALP, OCN, CTR, CTSK, NFATc1, and TRAF6 by 45%, 500%, 25%, 40%, and 40%, respectively, and BALP level by 5-fold, while increasing OPG level by 3-fold after 12 weeks.	[98]
Sarsasapogenin (**11**)	*Anemarrhena asphodeloides* Bunge	LPS-induced osteolysis in mouse	Prevented LPS-induced bone loss in a dose-dependent manner at 5–10 mg/kg/d for one week.	[99]
		RANKL-induced OCG in BMMs	Inhibited RANKL-induced OCG by suppressing the gene expression of CFos, NFATc1, MAPK, F-actin, Nfatc1, ACP5, and CTSK by over 60% each at 4 μM.	
Kavain (**12**)	*Piper methysticum*	OVX mouse	Inhibited bone loss and OCG by 50% and elevated protein levels of BVD and TN at 10 mg/kg i.p., 3x/week for 6 weeks.	[100]
		MMCs, MCPs and BMMs	Reduced the expression of NFATc1, MMP-9, ACP5, Atp6v0d2, integrin β3, JNK, p38, ERK, and CTSK, and Ca2+ oscillation generally by more than 30% each at 20 μM on day 6.	
Helvolic acid (**13**)	*Aspergillus fumigatus*	RANKL-induced OCG in BMMs	Reduced OCs by 60% through reduced expression of NFATc1, MAPK, MMP-9, ACP5, and CTSK, and Ca^2+^ oscillation by over 80% at 10 μM for five days	[101]
Berberine (**14**)	*Coptis chinensis*	LPS-generated bone loss in mice	Dose-dependently abrogated LPS-engineered bone loss in mice between 50 and 150 mg/kg/d for 10 days.	[102]
		RANKL-induced OCG in BMMs	At 5 μM, inhibited OCG via reduction in NFATc1, CFos, p38, ERK, TRAP, OSCAR, and ATP6v0d2 expression and F-actin ring formation by more than 80% and increased IκBα expression by over 60% after 7 days of treatment.	
Asperpyrone A (**15**)	*Aspergillus niger*	RANKL-induced OCG in BMMs	Prevented OCG via decrease in gene expression of NFATc1, CFos, CTSK, MAPK, and NF-κB by 66%, 66%, 40%, and 33%, respectively, and ROS production by 75% at 10 μmol/L for 6 days.	[103]
Asiaticoside (**16**)	*Centella asiatica* (L.) Urb.	RANKL-induced OCG in BMMs	Reduced OCG by 50% at 20 μM via suppression of NFATc1, CFos, MAPKs, MMP-9, ACP5, CTSK, NF-κB, TRAP, and ATP6v0d2 expression, and Ca^2+^ oscillation by over 30% each at 20 μM after 3 days.	[104]
Arctiin (**17**)	*Arctium lappa* L.	OVX mice	At 10 mg/kg/48 h, i.p., arctiin prevented bone loss and RANKL-induced OCG through the reduction in expression of NFATc1, ACP5, CFos, TRAP, ERK, JNK, MMP-9, and CTSK, and Ca^2+^ oscillation and F-actin ring formation by over 25% each at 20 μM for six days.	[105]
		RANKL-induced OCG in BMMs		
Madecassoside (**18**)	*Centella asiatica* (L.) Urb	OVX mice	Prevented bone loss and increased BMD, trabecular number, Oc.S/BS, and N.Oc/BS by over 25% each 10 mg/kg/48 h, i.p. for 6 days.	[106]
		RANKL-induced OCG in BMMs	At 10 μmol/L, madecassoside reduced OCs by over 15% via suppression of NFATc1, CFos, ACP5 TRAP, ERK, NF-κB, JNK, MMP-9, CTSK, and integrin β3 expression, and Ca^2+^ oscillation and F-actin ring formation by more than 30% while upregulating IκBα expression by 50% for 3–6 days.	
Acteoside (**19**)	*Rehmannia glutinosa*	OVX mice	Prevented bone loss in mice and decreased serum levels of TNF-α, IL-1β, TRAP, and OCN, and Ca^2+^ oscillation by more than 30% at 1 mM/72 h for eight weeks.	[107]
		RANKL-induced OCG in BMMs and MMCs	It also attenuated OC by over 80% at 20 µM through reduction in expression of NF-kB, TNF-α, IL-1β, TRAP, Ca^2+^ oscillation, CFos, NFATc1, ERK, and JNK by over 70% each after 24 h.	
Isoliquiritigenin (**20**)	*Glycyrrhiza glabra* L.	LPS-induced mice	Inhibited LPS-induced bone erosion by 90% via reduction in F-actin ring formation, lacunar resorption pits formation, and OcS/BS by over 30% after 8 weeks of treatment.	[94]
		RANKL-induced OCG in BMMs and MMCs	Inhibited OC via suppression of gene expression profiles of NF-kB, TRAF6, MAPKs, AP-1, CTSK, ERK, NFATc1, CFos, TRAP, CatK, MMP-9, and OSCAR by over 30% each and upregulated IκBα, OPG, and osteoblastic marker genes by over 30% at 50 μM for 72 h.	
Neogambogic acid (**21**)	*Garcinia hanburyi*	RANKL-induced OCG in BMMs	Neogambogic acid inhibited RANKL-induced OCG by 87.5% via the suppression of gene expression of NF-kB, JNK, TRAPs, CTR, CTSK, and NFATc1 by 60%, 40%, 50%, and 40%, respectively, at 0.4 μg/mL for 6 days.	[108]
Echinocystic acid (**22**)	*Eclipta prostrata*	OVX mouse model	Inhibited loss in bone mass, strength, and density, and reduced serum OCN, ALP, and deoxypyridinoline levels by 60%, 25%, and 35%, respectively, and improved bone histology with concurrent downregulation of expression profiles of IL-1β and TNF-α by 40% and 30%, respectively, in bone tissues at 15 mg/kg/d for 12 weeks.	[109]
Cimiracemate A (**23**)	*Cimicifuga racemose* L.	Glucocorticoid-induced OP in rats	Inhibited bone loss as characterized by increase in levels and gene expression profiles of TN, TT, BV/TV, HDL, OCN, and OPG by 50%, 40%, 150%, 136%, 130%, and 150%, respectively, with concurrent reduction in serum level and gene expression of TS, SIM, LDL, TC, ApoA1, ApoB, TAG, CTX, IL-1β, TNF-α, TRAP, RANK, and RANKL between 26% and 50%, respectively, at 10 mg/kg for 6 weeks.	[96]
Hesperetin (**24**)	Citrus fruit	OVX mice	Inhibited bone resorption by increasing the levels of TN, TT, BV/TV, and ALP by 35%, 17%, 125%, and 40%, respectively, with concurrent reductions in levels of TS, ALP, OC, TRAP, and CTX by over 17% each at 11 mg/kg i.p., 3x/week for 8 weeks. Inhibited OC by suppressing gene expression of CTSK, JNK, ERK, NFATc1, CFos, TRAP, ACP5, CTX, MMP-9, NF-kB, TRAF6, and Irf-3, by over 22% in each at 60 μM for 10 days.	[110]
		RANKL-induced OCG in BMCs, BMMs, and splenocytes		
Galangin (**25**)	*Alpinia officinarum*	LPS-induced osteolysis in mice	Inhibited bone loss by reducing F-actin ring formation and resorption indices by 90% and 75%, respectively, and increasing TN, TT, and BV/TV by 5% each at 10 mg/kg for 7 days.	[111]
		RANKL-induced OCG in BMCs	Inhibited OC differentiation by reducing the gene expression of TRAP, CTSK, p38, ERK, NFATc1, and CFos by 40%, 55%, 57%, 50%, and 75%, respectively, while increasing IκBα and p65 gene expression profiles at 12 μmol/L for 6 days.	
4-hydroxy-7-methoxycoumarin (**26**)	*Cynara scolymus* L.	RANKL-induced OCG in MMCs	Inhibited OCG via promotion of gene expression of IκBα by 10-fold while downregulating the expression of TNF-α, IL-1β, IL-6, NO, PGE_2_, iNOS, COX-2; NF-kB, ERK, and JNK by over 45% each at 1 mM.	[112]
Hypericin (**27**)	*Hypericum perforatum* L.	Titanium particle-induced osteolysis in mice	Inhibited bone loss reducing bone level of NP and PP, with concomitant increases in TN, TT, and BV/TV levels by at least 85% at 15 μg/kg for 10 days.	[113]
		RANKL-induced OCG in BMCs and BMMs	Inhibited OC formation by 88% by reducing the gene expression of resorption indices, NFATc1, CFos, ERK, MMP-9, ACP5, CTSK, NF-κB, CTR, TRAP, and ATP6v0d2, and F-actin ring formation and Ca^2+^ oscillation by over 65% each at 1.2 μM for 2 days.	
Arctigenin (**28**)	*Arctium lappa* L.	LPS-induced bone erosion in mice	Inhibited bone loss by increasing bone levels of TN, TT, and BV/TV by 85% each, while decreasing the levels of F-actin ring formation, resorption indices, and OC over 50% at 10 mg/kg for one week.	[114]
		RANKL-induced OCG in BMMs	Inhibited OCG by suppressing the gene expression of Syk, PLCγ2, Gab2, ERK, NFATc1, CFos, TRAF6, c-Src, and CTSK by 30% and above at 10 μM.	
Rhaponticin (**29**)	*Rheum undulatum* L.	RANKL-induced OCG in BMMs	Inhibited OC formation by increasing the expression of IκBα b 50% while downregulating the expression of F-actin ring formation, OC, resorption indices, NFATc1, CFos, CTSK, ATP6v0d2, and integrin β3, and ROS production, and Ca^2+^ oscillation by over 30% at 50 μM for 6 days.	[115]

MMCs—RAW264.7 mouse macrophages; MCPs—MC3T3-E1 pre-osteoblasts; BMMs—primary murine-derived bone marrow macrophages; ROS—reactive oxygen species; AP-1—activator protein 1; ATP6v0d2—ATPase Hþ transporting V0 subunit d2; CTR—calcitonin receptor, OSCAR—osteoclast-associated receptor; TRAF6—tumor necrosis factor (TNF) receptor-associated factor-6; N.Oc/BS—OC number per bone surface; Oc.S/BS—osteoclast surface per bone surface; BMD—bone mineral density; TN—trabecular number; TT—trabecular thickness; TS—trabecular separation/space; SIM—structure model index; BV/TV—bone volume/total volume; BVD—bone volume density; CTX—collagen type I fragments; LDL—low-density lipoproteins; high-density lipoproteins; TC—total cholesterol; Apo—apolipoprotein; TAG—triacylglycerol; GSR—glutathione S-reductase; CAT—catalase; NQO1, Nrf2/Keap1—NF-E2-related factor 2/Kelch-like ECH-associated protein-1; ARE—antioxidant response element; p.o.—per oral injection; i.p.—intraperitoneal injection; Sc—subcutaneous injection; PP—percentage porosity; NP—number of porosity; OCN—osteocalcin.

**Table 2:** Natural compounds with osteoprotective properties via BMP2-Wnt/β-catenin and PI3K/Akt/GSK3β signaling pathways.

Natural Compound	Source	Study Model	Specific Therapeutic Activity	Ref.
Icariin (**31**)	*Herba Epimedii*	Hydrogen peroxide-induced MC3T3-E1 cell oxidative damage	Alleviated H_2_O_2_-induced cytotoxicity and inhibitory osteogenic effect and promoted osteogenesis by increasing the levels of GSH and SOD by 140% and 120%, respectively, and decreasing MDA level by 47%, while the gene expression profiles of ALP, RUNX2-2, OSX, β-catenin, and cyclin D1 increased by 167–233%, at 0.1 μM for 48 h. Promoted cell proliferation and improved osteogenic differentiation and calcium deposit by enhancing the expression and levels of RUNX2, DEC1, BV/TV, p-GSK3β p-Akt, and Tb.Th by 100%, 90%, 11%, and 10%, respectively, at 1 nM. Protected against glucocorticoid-induced osteoporosis by enhancing gene expression of DEC1, ALP, and RUNX2 by 50%, 40%, and 50%, respectively, in bone tissues and mineralization by ~50% at 250 mg/kg for 60 days.	[128]
		SaoS-2 cells and mice		
Chlorogenic acid (**32**)	*Flos Lonicerae Japonicae*, *Eucommia Ulmoides*	MC3T3-E1 cells	Reversed inhibition of cell viability and reduced H_2_O_2_-induced oxidative damage and suppressed apoptosis (caspase-3 level) by ~44%, while increasing gene expression of HO-1, Nrf-2, and p-Akt by over 2-fold each at 400 μM for 48 h.	[160]
Albiflorin (**34**)	*Paeonia lactiflora*	MC3T3-E1 cells	Induced and promoted OBG by 40%, and improved bone union by upregulating the gene expression of RUNX2, ALP, OCN, OSN, OSX, BSP, OPN, BMP-2, p-Smad1/5, Wnt10b, β-catenin, LRP5, LRP6, Dvl2, and cyclin D1 by 50%, 20%, 40%, 50%, 25%, 50%, 33%, 22.2%, and 50%, respectively, in bone tissues at 10 mg/kg for 3 weeks.	[144]
Quercetin (**36**)	*Allium cepa*	MC3T3-E1 cells	Reversed LPS-induced suppression of bone mineralization and cell viability and reduced LPS-induced osteoblast apoptosis by 53% and totally restored inhibited osteoblast differentiation by increasing OSX, ALP, RUNX2, OCN, Wnt3, β-catenin, p-ERK1/2, Bcl-2, and Bcl-XL gene expression by 300%, 600%, 500%, 320%, 57%, 200%, and 150%, respectively, while suppressing gene expression of caspase-3, BAX, Cyt-C, MAPK, ERK, p-p38, and GSK3β by over 32% each at 10–50 μM for 7 days.	[145]
Polydatin (**37**)	*Polygonum* *Cuspidatum*	hBMSCs	Improved the proliferation and osteogenic differentiation by 183% at 10 μM and induced the formation of calcium nodules through the elevated expression of ALP, RUNX2, OPN, DLX5, OCN, COL1A1, BMP2, β-catenin, LEF1, TCF7, c-jun, c-myc, and cyclin D between 30% and 475% at 30 μM for 14 days.	[134]
3,5-dicaffeoyl-epi-quinic acid (**38**)	*Atriplex gmelinii*	hBM-MSCs	Enhanced osteoblast differentiation of osteo-induced hBM-MSCs by upregulating ALP, OCN, RUNX2, BMP2, Wnt 10a, p-p38, p-ERK, p-JNK, β-catenin, and Smad1/5 gene expression between 20% and 180%, while suppressing the expression of PPARγs and C/EBPα, and SREBP1c by 27% and 10%, respectively, at 10 mg/kg for 7 days.	[140]
Epigallocatechin (**40**)	Green tea	hPDLCs and hAOBs	Increased proliferation, differentiation, and osteoblast formation in hAOBs and hPDLCs by 100% and 150% via enhancement of ALP, RUNX2, BMP2, OSX, OCN, p-P13K, and p-Akt gene expression by 40%, 200%, 400%, 600%, 400%, 150%, and 150%, respectively, in hAOBs and 133%, 600%, 500%, 400%, 300%, 180%, and 160%, respectively, in hPDLCs at 10 μM for 5 days.	[157]
Sesamin (**42**)	*Sesamum indicum*	Rat primary osteoblasts	Inhibited ROS-induced osteoblast apoptosis by 75% by upregulating p-AKT and Bcl-2 gene expression by 300% and 400%, respectively, while downregulating the expression of Bax, caspase-3, and ROS production by 63%, 67%, and 70%, respectively, at 5–20 µM for 24 h.	[152]
Garcinol (**43**)	*Garcinia indica*	RANKL-induced OCG in BMMs	Attenuated bone resorption and Suppressed osteoclast differentiation through the downregulation of TRAP, CTSK, NFATc1, V-ATPase d2, p-ERK, p-p38, p-JNK, p-p65, p-Akt, C-jun, and Cfos gene expression and F-actin ring formation by 81%, 75%, 75%, 75%, 90%, 55%, 40%, 50%, 100%, 50%, 50%, and 69%, respectively, at 32 μM for 7 days. Prevented bone loss and RANKL-induced OCG by improving BV/TV by 2-fold and suppressing porosity and TRAP-positive OCs/BS by 54% and 71%, respectively, at 1 and 5 mg/kg for 7 days.	[159]
		LPS-induced calvarial osteolysis mice		
Macrolactins F (**44**)	*Bacillus amyloliquefaciens* Deep sea bacterium	RANKL-induced OCG in primary BMMs	Inhibited RANKL-induced osteoclast formation, and inhibited bone resorption activity in BMMs via increased mineralization by 80% at 20 μM for 5 days. Decreased the expression profiles of c-myc, RANK, TRAP, NFATc1, CTSK, MMP-9, CFos, p-JNK, p-p38, and p-Akt, and F-actin ring formation by 90% and promoted pre-osteoblast cell differentiation, and upregulated ALP, NF-kB, RUNX2, osterix, Smad4, BMP-2 COL1A1, OPN, and OCN levels by over 50% at 0–40 μM for 21 days.	[161]
		MC3T3-E1 cells		
Vinpocetine	*Phyllostachys pubescens*	DEX-induced rat osteoblasts apoptosis and ONFH	Suppressed DEX-induced over-regulation of ROS level and inhibited DEX-induced apoptosis in osteoblast by 50% each and protected against ONFH in rats. Increased gene expression of BV/TV, TN, p-Akt, Bcl-xl, and Bcl2 by 40%, 33.3%, 120%, 50%, and 50%, respectively, while suppressing ROS production and JNK, caspase3, and Bax gene expression by over 30% at 10 μM for 24–48 h.	[154]

Abbreviations: hBM-MSCs—bone marrow-derived human mesenchymal stromal cells; ALP—alkaline phosphatase; OCN—osteocalcin; RUNX2—runt-related transcription factor; BMP—bone morphogenetic protein; JNK—c-Jun N-terminal kinase; MAPK—mitogen-activated protein kinase; PPAR—peroxisome proliferator-activated receptor; C/EBP—CCAAT-enhancer-binding protein; SREBP—sterol regulatory element-binding protein Oc.S/BS—osteoclast surface per bone surface; BV/TV—bone volume/total volume; Nrf2—NF-E2-related factor 2; OPN—osteopontin; ERK—extracellular receptor kinase; hBMSCs—human bone mesenchymal stem cells; TCF—T-cell factor; LEF—lymphoid enhancing factor; OSX—osterix; Cyt-C—cytochrome c; GSK—glycogen synthase kinase; NFATc1—nuclear factor of activated T-cells cytoplasmic 1; ERK1/2—extracellular signal-regulated kinases 1/2; AKT—protein kinase B; DLX5—Distal-less homeobox 5; Wnt—wingless/integrated; c-myc—cellular myc; Bcl-2—B-cell lymphoma; Bcl-xl—B-cell lymphoma extra-large; BAX—Bcl-2-like protein 4; LRP—low-density lipoprotein receptor-related protein; BSP—bone sialoprotein; Dvl-2—disheveled 2; DEC1—deleted in esophageal cancer 1.

**Table 3:** Natural products targeting the calcium signaling axis with potent relevance to bone health.

Natural Product	Source	Cell Line/Animal Model	Specific Therapeutic Activity	Ref.
Ligustroflavone (**46**)	*Ligustrum lucidum*	HEK-293 cells and diabetic mice	Reduced CaSR and serum PTH level by 42.95% while BV/TV, serum Ca, and bone Ca levels were increased by 88.9%, 7.1%, and 41.67%, respectively, at 10^−10^–10^−4^ M for 2–24 h.	[174]
Leu-Arg-Trp	Pea protein	Pre-osteoblast MC3T3-E1 cells	Increased COL1, ALP, RUNX2-2, and OPG levels, and cell proliferation while inhibiting bone resorption by over 20% each at 50 µM in cell culture for 24 h.	[178]
Trp-His	Pea protein	Ca^2+^-encapsulation fluorescence assay and in silico molecular dynamic simulation	Reduced CaMKII level by 31.67%, and Hills binding coefficient of Ca^2+^ to CaM from 2.81 to 1.92 (in silico approach).	[176]
Crocin (**47**)	*Crocus sativus*	Rat cardiomyocytes	Inhibited L-type Ca^2+^-gated channel while increasing Ca^2+^-signaling axis by more than 47% each at 1–300 µM for 12 min.	[180]
Urolithin C (**48**)	*Crocus sativus*	INS-1 beta-cells	Increased intracellular calcium uptake by over 50%.	[179]
Sclareol (**49**)	*Salvia sclarea*	Oxytocin-induced uterine hypercontraction dysmenorrhea model	Promoted Ca^2+^–MLCK–MLC20 signaling cascade by 50% at 10–100 µM every 10 min.	[181]
γ-Glutamyl valine (γ-EV)	Edible beans	HAoECs	Increased CaSR and β-arrestin by ~40 and 46%, respectively, while inhibiting GIT inflammation by over 30% at 0.01–1 mM for 2 h.	[173]
Curcumin (**50**) and Alpha interacting domain of L-type Ca^2+^ channel packaged in a poly(glycidyl methacrylate) (PGMA) nanoparticles	*Crocus sativus*	Rat cardiomyocytes	Decreased L-type Ca^2+^-gated channel and release of superoxides from 53% to 7.32% for 0.2–20 μM for 20 min.	[182]

OPG—osteoprotegerin; COL1—type 1 collagen; CaMKII—Ca^2+^/calmodulin (CaM)-dependent protein kinases II; HAoECs—human aortic endothelial cells; GIT—gastro-intestinal tract.

**Table 4:** Clinical studies on humans illustrating the bone health-improving effects of natural products.

Natural Products/Sources	Clinical Trial Design	Number of Individuals That Started/Completed the Trial	Parameter Investigated and Outcomes (Bone Formation Marker (BFM) and Bone Resorption Marker (BRM))	Outcome	Ref.
Polyphenols/Green tea	24-week (daily treatment) randomized and placebo-controlled interventional trial	Postmenopausal women with osteopenia (171 started and 150 completed)	(a) Change from baseline (100%) in a ratio of BFM (BSAP) to BRM (TRAP)	(a) 103.6 ± 2.9%	[198]
Reconstituted dairy products enriched with Ca, Vit D, Vit K, Vit C, Zn, Mg, Leu, and probiotic (Lactobacillus plantarum)	24-week (daily treatment) randomized, parallel, double-blind clinical trial with two intervention groups	Both healthy menopausal women with risk of osteoporosis and those with untreated osteopenia (78 started and completed)	(a) Bone mass of the EG vs. CG significantly (*p <* 0.05) increased (b) BMD was maintained in the EG and depleted in the CG (c) BFM (P1NP) significantly (*p <* 0.05) increased in the EG compared to the CG (d) BRM (CTx) significantly (*p <* 0.05) decreased in the EG compared to the CG	(a) 0.01 ± 0.03 (EG) vs. −0.01 ± 0.03 kg (CG) (b) Data not available (c) 13.19 ± 25.17 (EG) vs. −4.21 ± 15.62 (CG) ng/mL (d) −0.05 ± 0.19 (EG) vs. 0.04 ± 0.14 (CG) ng/mL	[199]
Hop rho iso-alpha acids (200 mg) and berberine sulphate trihydrate (100 mg)	14-week, single-blinded, 2-arm placebo-controlled pilot study	Postmenopausal women on Mediterranean low-glycemic diet and limited aerobic exercise (33 started and 32 completed)	(a) BRM (osteocalcin) significantly (*p <* 0.01) decreased in EG compared to CG (b) Serum 25(OH)D-significantly increased in the ED compared to the control (c) Serum IGF-1 was significantly higher in the EG compared to the control	(a) −31% (EG) vs. +19% (CG) (b) 13% (EG) vs. −25% (CG) (c) Data not shown	[200]
Hop rho iso-alpha acids (200 mg) and berberine sulphate trihydrate (100 mg)	14-week, randomized trial, 2-arm placebo-controlled interventional study	Postmenopausal women on Mediterranean low-glycemic diet and limited aerobic exercise (51 started and 45 completed)	(a) BRM (osteocalcin) significantly (*p <* 0.01) decreased in EG compared to CG (b) Serum 25(OH)D significantly increased in the ED compared to the control (c) Serum IGF-1 was non-significantly higher in the EG compared to the control	(a) −25% (EG) vs. + 21% (CG) (b) 23% (EG) vs. −12% decrease (c) Data not available	[201]
Red clover extract (RCE) rich in Isoflavone aglycones and probiotics	12-month, double-blind, parallel design, placebo-controlled, randomized controlled trial	Postmenopausal osteopenic women supplemented with calcium (1200 mg/d), magnesium (550 mg/d), and calcitriol (25 μg/d); 87 started and 78 completed	(a) BMD loss was significantly reduced in the EG compared to the CG in the following regions L2-L4 lumbar spine vertebra (*p* < 0.05) femoral neck (*p* < 0.01) trochanter (*p* < 0.01) (b) BRF (CT1CLCT) was significantly reduced in the EC compared to the CG	(a) −0.99% (EG) vs. −2.2% (CG) −1.04% (EG) vs. −3.05% (CG) −0.67% (EG) vs. −2.79 (CG) (b) −9.40% (EG) vs. −6.76% (CG)	[202]
Collagen peptide from pork skin and bovine bone	13 weeks, double-blind, placebo-controlled, randomized trial (5 g investigational product or placebo dissolved in 250 mL water or milk administered orally twice daily)	30 patients (male and female) with knee osteoarthritis (VAS score ≥ 4 and KLG—2 to 4)	(a) Questionnaire assessment of pain, stiffness, and physical function using the WOMAC, VAS, and QOL score. The outcome was reported as the percentage number of patients with 20 points or more reduction in WOMAC score from the baseline 40 mm or more reduction in the VAS score from the baseline on 100 mm scale 20 points or more reduction in the QOL score from the baseline	(a) Significant reduction in WOMAC score at visit 7 (*p* < 0.05 at visit 4, *p* < 0.01 at visit 5–7) (b) Significant reduction in VAS score at visit 7 (*p* < 0.01 at visit 4–7) (c) Significant reduction in QOL score at visit (*p* < 0.01 at visit 4–7)	[203]
Prenylflavonoids from *Epimedium* spp.	6 weeks randomized, double-blinded clinical trial prenylflavonoid extract (740 mg daily) or placebo daily for 6 weeks	Healthy postmenopausal women administered prenylflavonoid extract (740 mg daily) or placebo daily (58 started and completed the trial)	(a) Adverse effects on administering prenylflavonoid capsule (b) Metabolites in the sera (c) C_max_ and AUC_0→__∞_ for metabolites (d) Increase in BFM (BSAP) in EG compared to the CG (e) Decrease in BRM (TRAF6) in the EG compared to the CG	(a) No adverse symptoms or changes in hepatic, hematological, and renal parameters (b) Desmethylicaritin, icaritin, and icariside II were detected in the sera (c) C_max_ and AUC_0→__∞_ for desmethylicaritin of 60.9 nM, and 157.9 nM ×day (d) Significant higher BSAP (*p* < 0.05) (e) Non-significantly (*p* = 0.068) lower level of TRAF6	[204]
Collagen peptide hydrolysate (Fortibone^®^)	3-month randomized, parallel assignment, interventional clinical trial (administered with 5 mg of collagen peptides)	51 postmenopausal women within the osteopenic T-score range of (−1.0 > T-score > −2.5) at either the lumbar spine (LS) or femur	(a) Non-significant increase in BMI, and decrease in OC of the EG compared to CG (b) Significant percentage decrease from baseline to 3 months in P1NP and CTX of the EG when compared to the control group	(a) BMI (*p* = 0.842) and OC (*p* = 0.578) (b) −13.1 ± 12.3 (EG) vs. −2.1 ± 12.6 (CG) (*p* = 0.011) for P1NPand −11.4 ± 24 (EG) vs. 3.5 ± 29.9 (CG) for CTx	[205]
Kefir-fermented milk peptides	6 months randomized, parallel, double-blind intervention study	Osteoporosis patients (male and female) of age between 55 and 70 years; 69 patients started and 40 completed the trial	(a) BMD after 6 months forspine, total hip, and hip femoral neck significantly increased in EG compared to CG (b) Serum level of β-CTX and OC	(a) Non-significant difference in spine (*p* = 0.909); total hip (*p* = 0.520); and femoral neck (*p* = 0.501) (b) Non-significant difference in OC and CTX (*p* = 0.325)	[206]
Dried plum (DP) (prune)	Randomized crossover trial (a group consuming ∼42 g DPs per day vs. a second group consuming ∼14 g DPs per day for 2 weeks)	Healthy, postmenopausal women (27 started and completed the trial)	(a) BRM (CTX) decrease in the group that consumed more DPs (∼42 g) after the crossover phase than the group that consumed fewer DPs (∼14 g)	(a) A significant decrease (*p* = 0.006)	[207]

BSAP—bone-specific alkaline phosphatase; TRAP—tartrate-resistant acid phosphatase; OC—osteocalcin; PTH—parathyroid hormone; EG—experimental group; CG—control group BMD—bone mineral density; P1NP—N-terminal propeptide of type I collagen; CTx—carbo-terminal telopeptide of type I collagen; 25(OH)D—25-hydroxyvitamin D; IGF-1—insulin-like growth factor I; N/A—not applicable or not available; CT1CLCT—collagen type 1 cross-linked C-telopeptide; VAS—visual analogue scale; KLG—Kellgren–Lawrence grade; WOMAC score—Western Ontario McMaster Universities Score; QOL score—quality of life score.

## Data Availability

Not applicable.

## Supplementary Materials

The supporting information can be downloaded at: https://www.mdpi.com/article/10.3390/ijms23158468/s1.
