# Supplementary material for: A Review on the Molecular Mechanisms of Action of Natural Products in Preventing Bone Diseases
Source: Int J Mol Sci. 2022 Jul 30;23(15):8468. doi: 10.3390/ijms23158468 (PMC9368769; doi:10.3390/ijms23158468)
Supplement: Supplementary file 1 [file ijms-23-08468-s001.zip › ijms-1831531-supplementary.pdf]

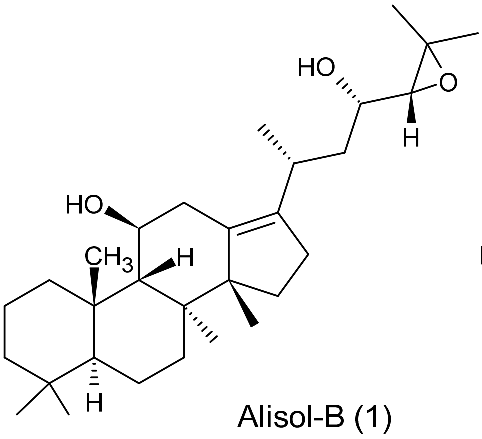

Alisol-B (1)

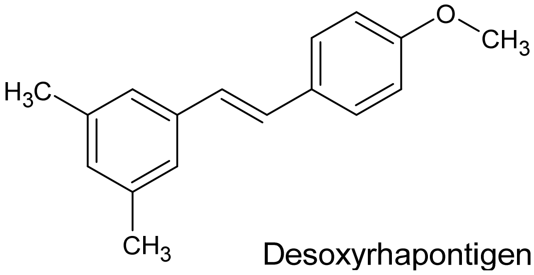

Desoxyrhapontigenin (2)

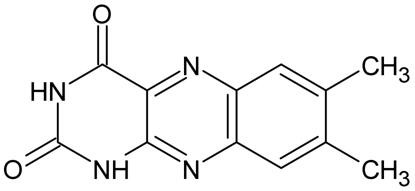

Lumichrome (3)

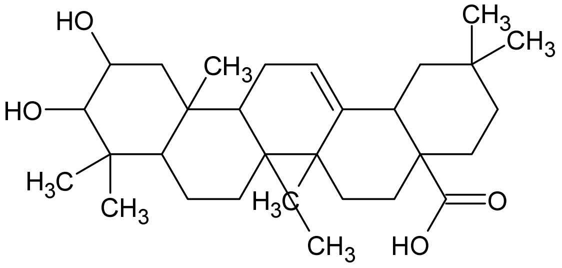

Maslinic acid (4)

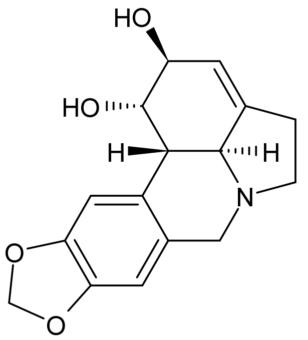

Lycorine (5)

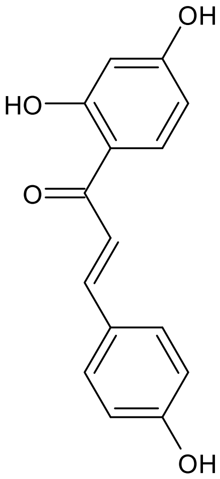

Isoliquiritigenin (6)

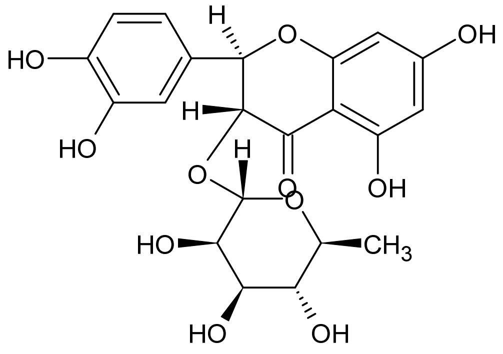

Astilbin (7)

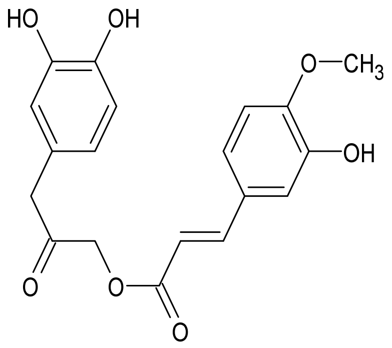

Cimiracemate A (8)

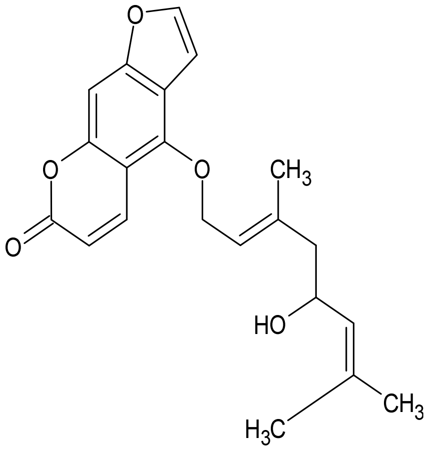

Notopterol (9)

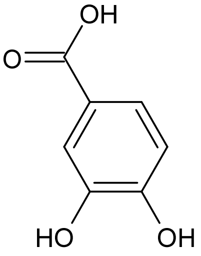

Protocatechuic acid (10)

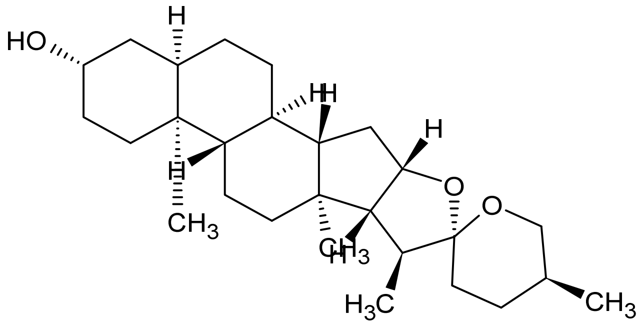

Sarsasapogenin (11)

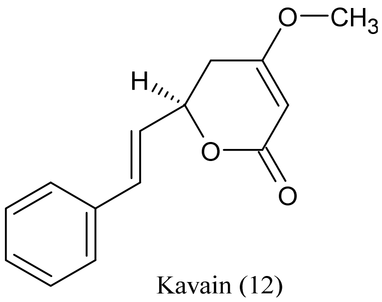

Kavain (12)

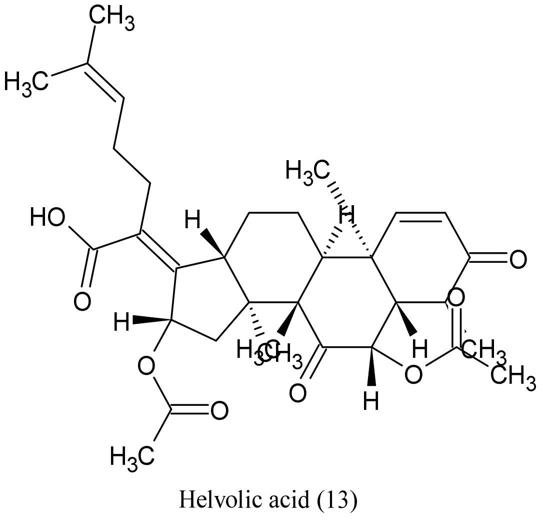

Helvolic acid (13)

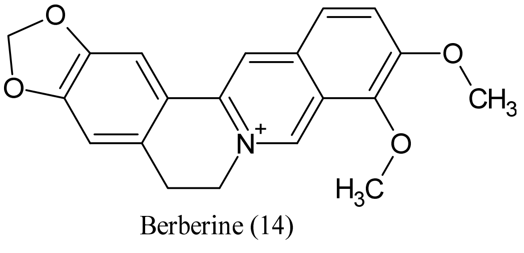

Berberine (14)

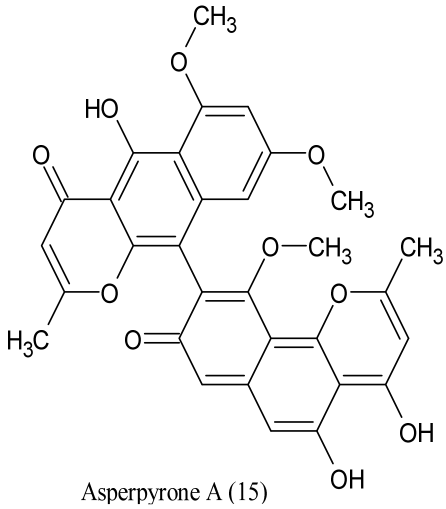

Asperpyrone A (15)

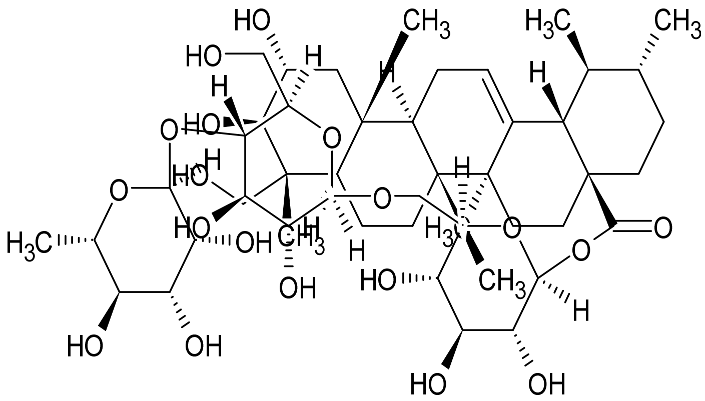
Asiaticoside (16)

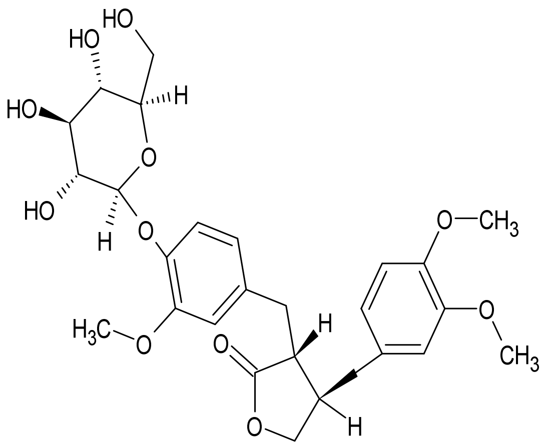
Arctiin (17)

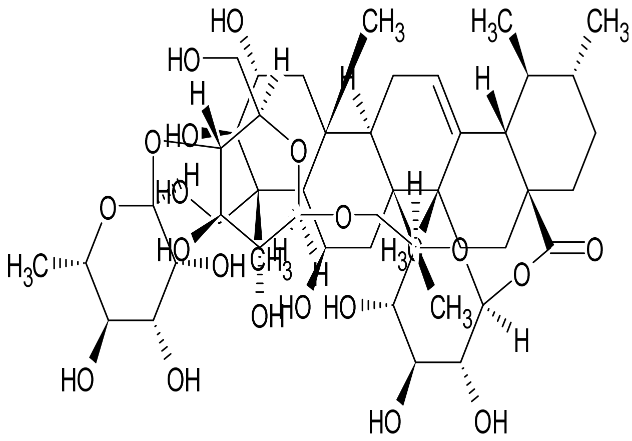
Madecassoside (18)

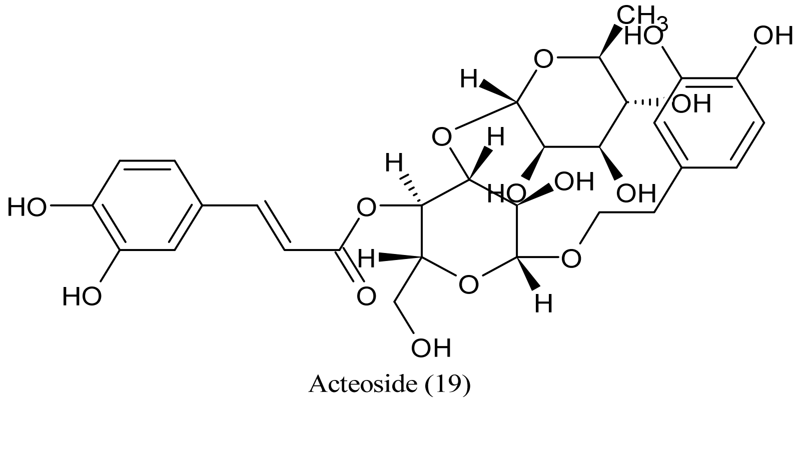
Acteoside (19)

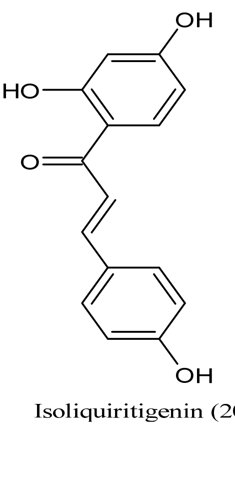

Isoliquiritigenin (20)

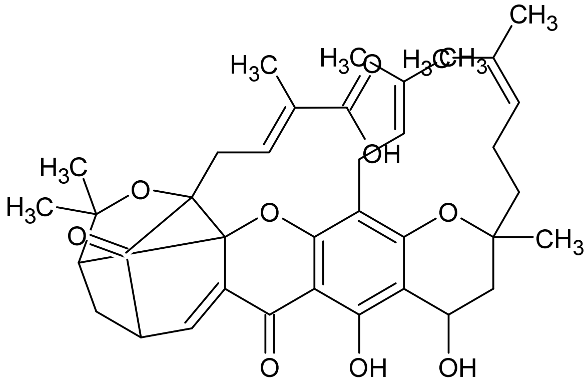

Neogambogic acid (21)

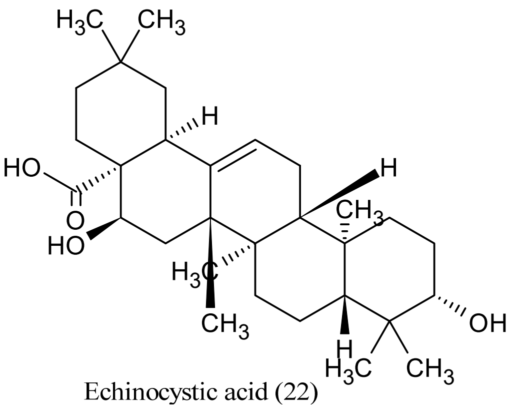

Echinocystic acid (22)

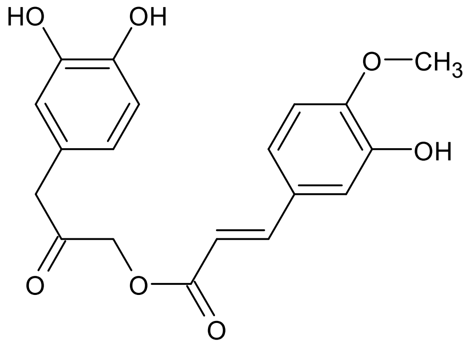

Cimiracemate A (23)

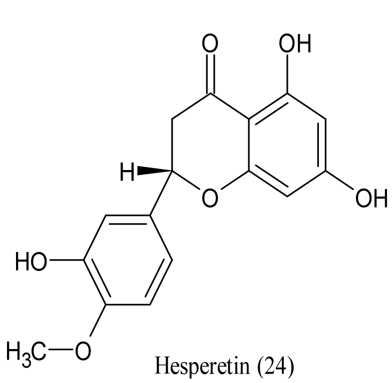

Hesperetin (24)

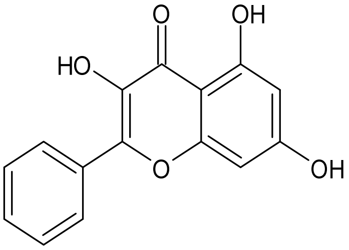

Galangin (25)

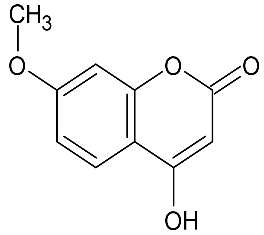

4-hydroxy-7-methoxycoumarin (26)

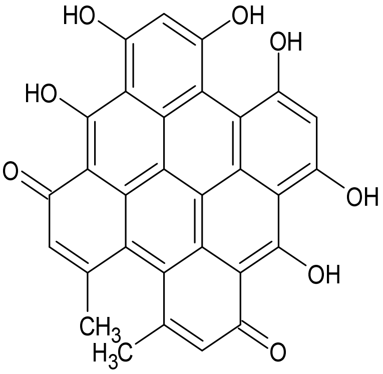

Hypericin (27)

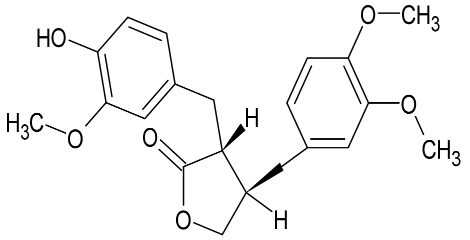

Arctigenin (28)

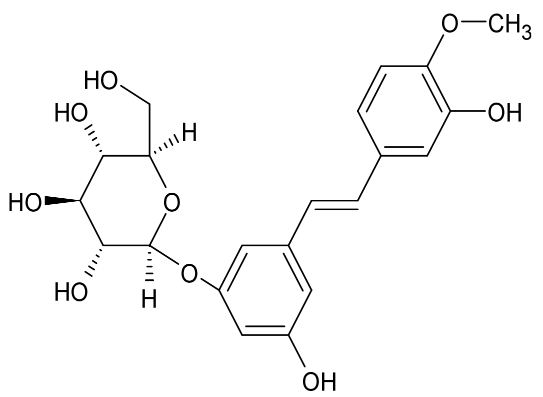

Rhaponticin (29)

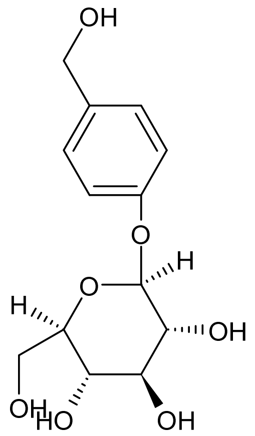

gastrodin (30)

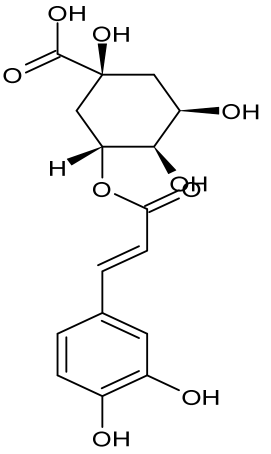

Chlorogenic acid (32)

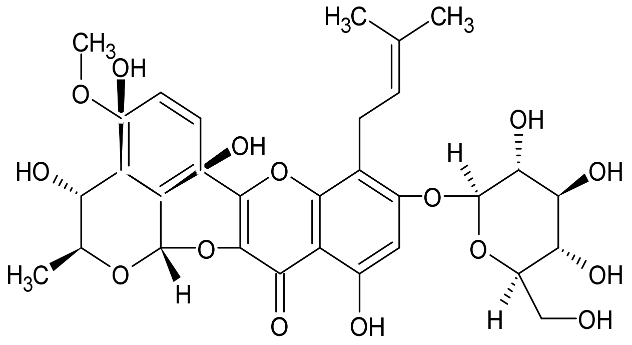

icariin (31)

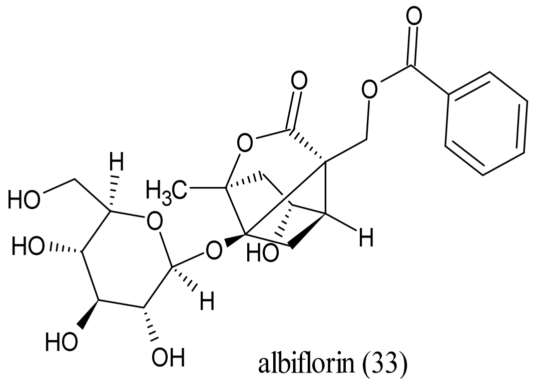

albiflorin (33)

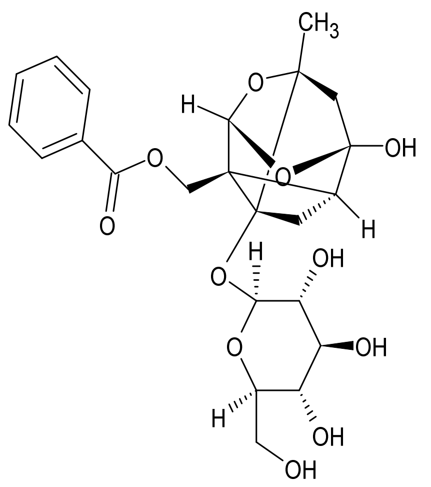

paeoniflorin (34)

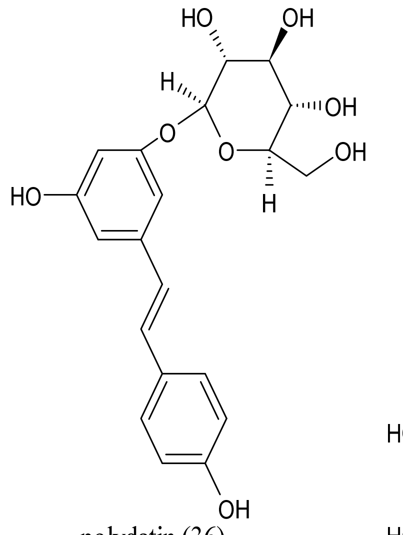

polydatin (36)

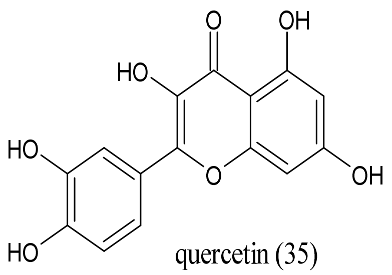

quercetin (35)

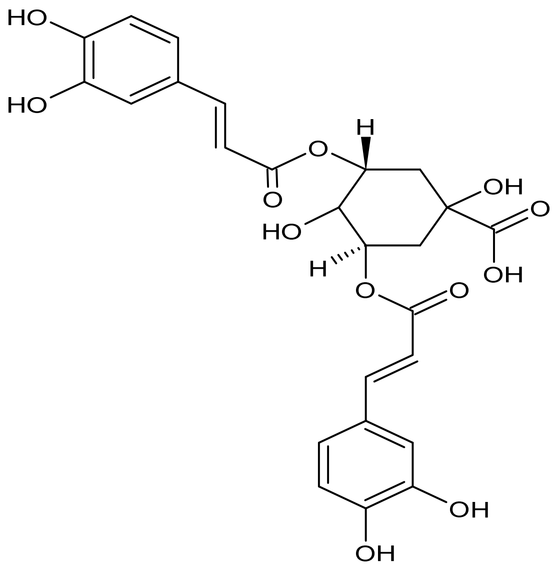

3,5-dicaffeoyl-epi-quinic acid (37)

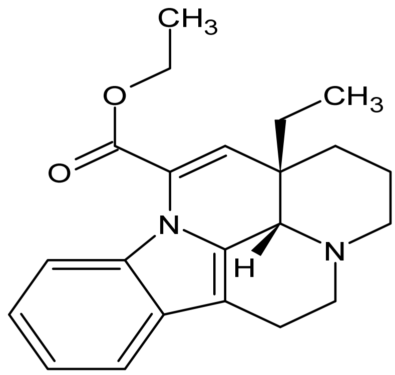

Vinpocetin (38)

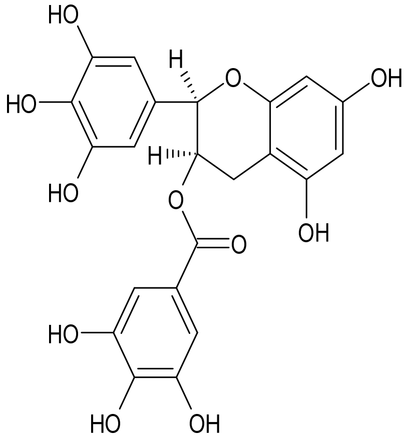

Epigallocatechin gallate (EGCG) (39)

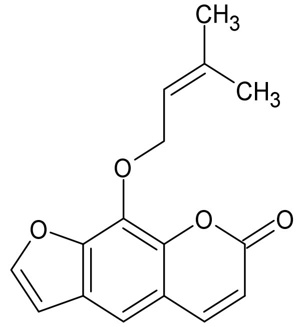

imperatorin (40)

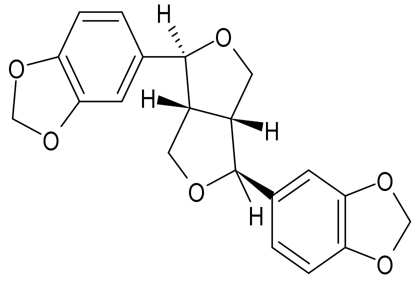

sesamin (41)

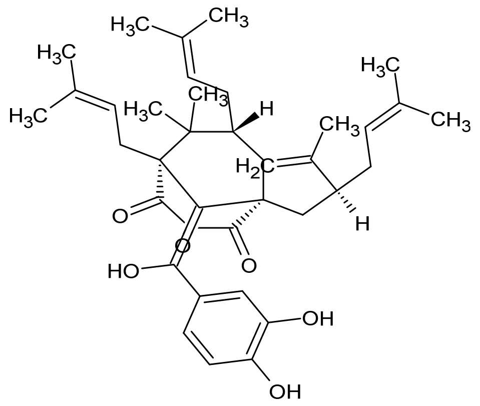

garcinol (42)

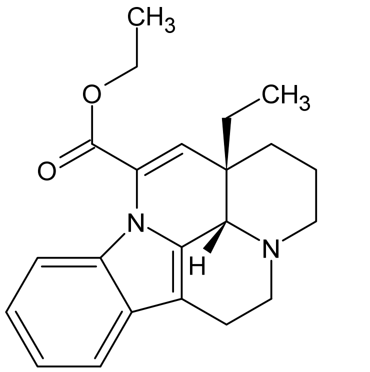

Macrolactins F (43)

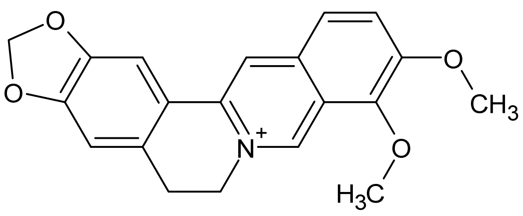

Berberine (44)

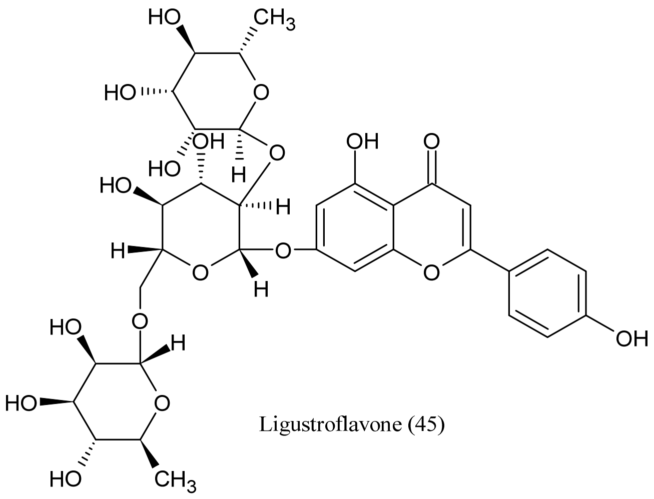

Ligustroflavone (45)

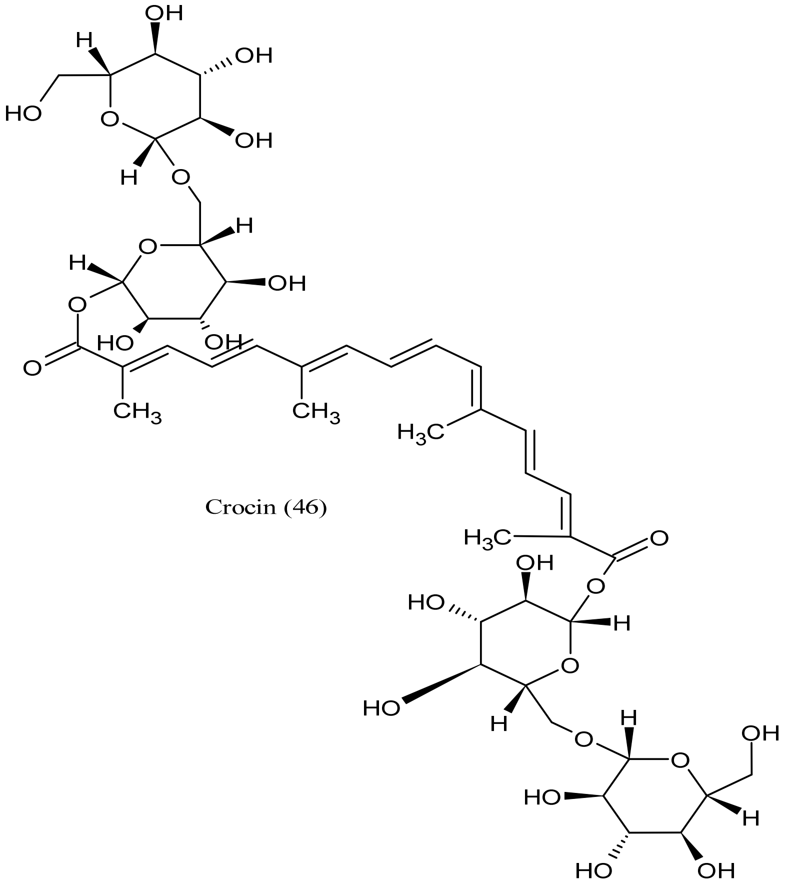

Crocin (46)

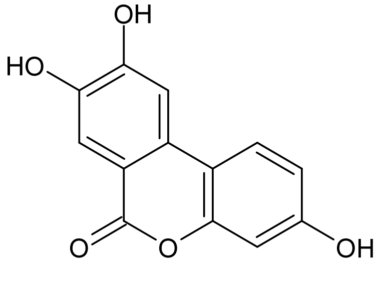

Urolithin C (47)

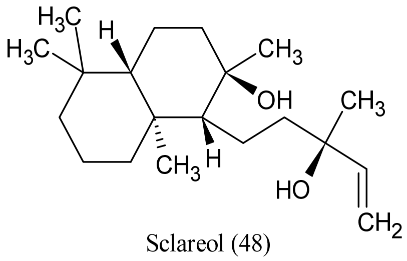

Sclareol (48)

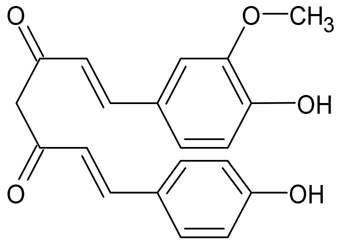

Curcumin (49)

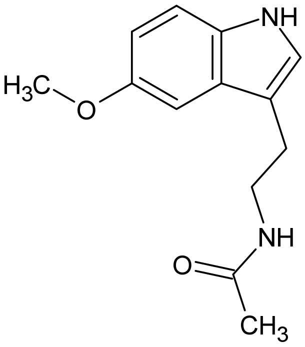

Melatonin (50)

Figure S1: Structures of selected natural products with osteoprotective properties

Abbreviations

AGE/RAGE (advanced glycated end-products/receptor)

Akt (protein kinase-B)

ALP (alkaline phosphatase)

AMPK (AMP-dependent kinase)

AP-1 =(activator protein 1)

Apc (adenomatosis polyposis coli)

Apo (apolipoprotein)

ARE (antioxidant response element)

ATP6v0d2 (ATPase Hþ transporting V0 subunit d2)

BAX (Bcl-2-associated X protein)

BDs (bone diseases)

BMD (bone mass density)

BMMs (primary murine-derived bone marrow macrophages)

BMP-2 (bone morphogenetic protein-2)

BSAP (bone-specific alkaline phosphatase)

BSP (bone sialoprotein)

BV/TV (bone volume/total volume)

BVD (bone volume density)

CaMKII (Ca2+/calmodulin (CaM)-dependent protein kinases)

CaSR (Calcium sensing receptors)

CAT (catalase)

Cbfa1(Core-binding factor alpha 1)

c-Fms (Colony-stimulating factor-1 receptor)

c-Src (proto-oncogene tyrosine-protein kinase)

CTR (calcitonin receptor)

CTSK (cathepsin K)

CTx (carbo-terminal telopeptide of type I collagen)

cyt c (cytochrome C)

DEX (dexamethasone)

DMP-1 (dentin matrix acidic phosphoprotein 1)

EGCG (Epigallocatechin gallate)

ERK (extracellular signal-regulated kinase)

FasL (Fas ligand)

FOXP3+ (forkhead box P3+)

GCS (γ-glutamylcysteine synthetase)

GSK3β (glycogen synthase kinase-3β)

GSR (glutathione S- reductase)

hAOBs (human alveolar osteoblaststs)

HDL (high-density lipoproteins)

HIF-1 (hypoxia-inducible factor 1)

HMGB1 (High mobility group box 1)

hPDLCs (human periodontal ligament cells)

i.p (intraperitoneal injection)

IFN-γ (interferon gamma)

IGF (insulin-like growth factor)

IL-1 (interleukin 1)

IL-17 (interleukin 17)

IL-1b/6 (interleukin 1 beta)

IL-6 (interleukin 6)

IκBα (kappa light polypeptide gene enhancer in B-cells inhibitor)

JNK (c-Jun N-terminal kinase)

Keap-1 (Kelch-like ECH-associated protein 1)

LDL (low-density lipoproteins)

MAPKs (mitogen-activated protein kinase)

MCPs (MC3T3-E1 preosteoblasts)

M-CSF (monocyte/macrophage colony stimulating factor)

MMCs (mouse macrophage cells)

MMP-9 (matrix metalloproteinase-9)

N.Oc (OC number per bone surface)

NFATc1 (nuclear factor of activated T Cells 1)

NF-κb (nuclear factor kappa B)

NOX-1/2 (NADPH oxidase ½)

NP (number of porosity)

NPs (Natural products)

NQO1 (NAD(P)H:quinone reductase)

NR1D (nuclear receptor subfamily 1, group D, members)

NR1D1 (nuclear receptor subfamily-1 group D member-1)

Nrf2 (nuclear factor erythroid 2–related factor 2)

OA (osteoarthritis)

Oc.S/BS (osteoclast surface per bone surface)

OCG (osteoclastogenesis)

OCN (osteocalcin)

OP (osteoporosis)

OPG (osteoprotegerin)

OSCAR (osteoclast-associated receptor)

OVX (ovariectomized)

p.o (per oral injection)

P1NP (N-terminal propeptide of type I collagen)

p38 MAPK (mitogen-activated protein kinase)

PGC-1α (peroxisome proliferator-activated receptor gamma coactivator 1-alpha)

PGE2 (prostaglandin E2)

PI3K (phosphoinositide 3-kinase)

PP (percentage porosity)

PTH (parathyroid hormone)

PUVA (Psoralen plus ultra violet radiation)

RA (Rheumatoid arthritis)

RANKL (receptor activation of NF-κB ligand)

ROS (reactive oxygen species)

SASP (senescence-associated secretory phenotype)

Sc (subcutaneous injection)

SERMs (selective estrogen receptor modulators)

SIM (structure model index)

sirt-1 (sirtuin-1)

SMAD (SMA (small worm phenotype); MAD (mothers against decapentaplegic))

SOST (sclerostin)

TAG (triacylglycerol)

TC (total cholesterol)

Th17 (T helper 17)

TN (trabecular number)

TNF-α (tumor necrosis factor-alpha)

TRAC (T Cell Receptor Alpha Constant)

TRAF6 (tumor necrosis factor (TNF) receptor-associated factor-6)

TRAF6TNF (receptor-associated factor-6)

TRAP (tartrate-resistant acid phosphatase)

TS (trabecular separation/space)

TT (trabecular thickness)

UVA (ultra violet radiation)

VDR (vitamin D receptor)

VEGF-A (Vascular endothelial growth factor alpha)

γ-EV (γ-Glutamyl valine)
